# High-resolution genomic surveillance elucidates a multilayered hierarchical transfer of resistance between WWTP- and human/animal-associated bacteria

**DOI:** 10.1186/s40168-021-01192-w

**Published:** 2022-01-25

**Authors:** You Che, Xiaoqing Xu, Yu Yang, Karel Břinda, William Hanage, Chao Yang, Tong Zhang

**Affiliations:** 1 Environmental Microbiome Engineering and Biotechnology Laboratory, Center for Environmental Engineering Research, Department of Civil Engineering, The University of Hong Kong, Pok Fu Lam, Hong Kong, China; 2 Center for Communicable Disease Dynamics, Department of Epidemiology, Harvard T. H. Chan School of Public Health, Harvard University, Boston, MA USA; 3 Department of Biomedical Informatics, Harvard Medical School, MA Boston, USA; 4 Key Laboratory of Molecular Microbiology and Technology for Ministry of Education, Nankai University, 300071 Tianjin, China

**Keywords:** Genomic epidemiology, MDR, Plasmidome, Insertion sequences, Nanopore, Horizontal gene transfer

## Abstract

**Background:**

Our interconnected world and the ability of bacteria to quickly swap antibiotic resistance genes (ARGs) make it particularly important to establish the epidemiological links of multidrug resistance (MDR) transfer between wastewater treatment plant (WWTP)- and human/animal-associated bacteria, under the One Health framework. However, evidence of ARGs exchange and potential factors that contribute to this transfer remain limited.

**Results:**

Here, by combining culture-based population genomics and genetic comparisons with publicly available datasets, we reconstructed the complete genomes of 82 multidrug-resistant isolates from WWTPs and found that most WWTP-associated isolates were genetically distinct from their closest human/animal-associated relatives currently available in the public database. Even in the minority of lineages that were closely related, WWTP-associated isolates were characterized by quite different plasmid compositions. We identified a high diversity of circular plasmids (264 in total, of which 141 were potentially novel), which served as the main source of resistance, and showed potential horizontal transfer of ARG-bearing plasmids between WWTP- and humans/animal-associated bacteria. Notably, the potentially transferred ARGs and virulence factors (VFs) with different genetic backgrounds were closely associated with flanking insertion sequences (ISs), suggesting the importance of synergy between plasmids and ISs in mediating a multilayered hierarchical transfer of MDR and potentiating the emergence of MDR-hypervirulent clones.

**Conclusion:**

Our findings advance the current efforts to establish potential epidemiological links of MDR transmission between WWTP- and human/animal-associated bacteria. Plasmids play an important role in mediating the transfer of ARGs and the IS-associated ARGs that are carried by conjugative plasmids should be prioritized to tackle the spread of resistance.

Video Abstract

**Supplementary Information:**

The online version contains supplementary material available at 10.1186/s40168-021-01192-w.

## Background

The emergence of extensive multidrug-resistant (MDR) bacteria has been recognized as a global health concern [1, 2], both in the clinical settings and in the environments, under the One Health framework. It is generally accepted that antimicrobial resistance (AMR) can no longer be addressed by simply studying the problem in healthcare settings since the health of human and environmental ecosystems is closely linked [3], and microorganisms from nonclinical ecosystems are often the original source of clinically important antimicrobial resistance genes (ARGs) [4, 5]. In particular, wastewater treatment plants (WWTPs) represent an important interface between humans/animals and the environment, where a complex and genetically diverse microbial community can exchange genetic materials associated with various adaptive traits, such as ARGs and virulence factors (VFs), potentially leading to the emergence of MDR-hypervirulent clones [6]. Additionally, WWTPs systems have not been intentionally designed to manage AMR concerns, a significant amount of bacteria carrying antibiotic resistance are continuously shed into receiving environmental reservoirs (e.g., rivers and recreational water) [7–9]. The discharged antibiotic-resistant bacteria derived from human and animal microbiomes are also the best candidates for transmitting resistance back to humans and animals [10, 11] through potential environmental exposures, such as wastewater reuse, ingestion, dermal contact, and the inhalation of various media influenced by WWTP sources [12–14]. Despite intense efforts to study the ARGs in WWTPs [15, 16], most of these studies have focused on the overall distribution and diversity of the resistome rather than on characterizing the repertoire of mobile resistance with high risk of dissemination, as well as investigating their relatedness among strains across different ecosystems and hence determining their likely transmission to pathogenic bacteria infecting humans and animals.

Importantly, determining the clinical impact of multidrug resistance requires a high-resolution phylogeny plus complete information regarding the adaptive accessory elements [17]. Although shotgun (short-read) genome and metagenome sequencing followed by assembly allows us to partially reconstruct the strain-level community structure and the genetic context of ARGs [18, 19], in the absence of complete genomes, we lack a thorough understanding of the evolutionary dynamics of resistance and the underlying mechanisms, such as the interactions between different mobile genetic elements (MGEs) in mediating the transfer of resistance. In addition, an analysis of the recent transfer of multidrug resistance and the associated MGEs across different genetic backgrounds would provide critical information on the persistence and stability of resistance and, above all, support the identification of the major contributors and the necessary genetic units that should be prioritized to tackle this critical public health threat. However, little is known about the epidemiological picture (e.g., sequence types (STs), ARGs and hypervirulence determinants) of MDR bacteria in WWTPs, as well as the potential links of MDR transmission between WWTP- and human/animal-associated bacteria.

Here, we present a genomic epidemiology study of 82 MDR bacteria isolated from three WWTPs in Hong Kong that were resistant to a combination of four antibiotics (ampicillin, kanamycin, tetracycline, and chloramphenicol). Using long-read (Nanopore sequencing) and short-read (Illumina) whole genome sequencing, we reconstructed the complete genomes of all these isolates, which included 264 circular plasmids. Through comparison with large collections of bacterial plasmids and genomes, we investigated the evolutionary relatedness among the WWTP MDR isolates and those of relevant species from humans/animals. Finally, we determined the major contributors that facilitate the dissemination of multidrug resistance and VFs across different genetic backgrounds.

## Methods

### MDR bacterial isolation from the influent and effluent of three WWTPs

Approximately 100 μL of influent from each of the three WWTPs in Hong Kong (Shatin: 22.407° N, 114.214° E; Shek Wu Hui: 22.510° N, 114.119° E; Stanley: 22.219° N, 114.210° E) was plated onto lysogeny broth (LB) agar supplemented with four categories of antibiotics (ampicillin: 100 mg/L; kanamycin: 50 mg/L; tetracycline: 20 mg/L; and chloramphenicol: 25 mg/L). These four antibiotics are representative of the major classes of antibiotics with relatively high resistance in WWTPs [20], plus their long history of usage, making them ideal candidates to study the dynamic distribution and transfer of their corresponding resistance genes. For each of the three effluent samples, bacteria were collected after filtering 1 L of each sample through a 0.45-μm cellulose nitrate membrane. The pellets on the membranes were suspended in 1 mL of LB medium, and approximately 100 μL of the suspension was plated on the same agar plate as described above. The plates were incubated for 12–18 h at 37 °C, and we then randomly selected at least 12 and 15 MDR isolates with reference to the morphology of the colonies from each of the influent and effluent, respectively (82 in total).

### DNA sequencing (Illumina and Nanopore) and genome assembly

Bacterial genomic DNA was extracted using a DNeasy PowerSoil Kit (Qiagen, Germany) following the manufacturer’s instructions. The extracted DNA was sequenced using the PE150 strategy (Illumina HiSeq4000) at Novogene Corporation (Beijing, China), and each sample yielded ~ 1 Gb of data. Raw fastq reads were processed for adapters and quality trimming as previously described [20]. For Nanopore sequencing, library preparation was performed using a rapid barcoding kit (SQK-RBK004) and sequenced with R9.4.1 flow cells on GridION. Raw Nanopore reads were base-called and demultiplexed using Guppy 3.0.3 (https://community.nanoporetech.com) to return separate fastq files. High-quality genome (including plasmids) of the isolates were generated by hybrid assembly of the Nanopore and Illumina reads using Unicycler [21]. Subculture and a new round of nanopore sequencing were performed for samples with contamination in the original illumina reads, and the genomes were assembled using Nanopore reads first and further polished with Medaka (https://github.com/nanoporetech/medaka) and Unicycler-polish with original illumina reads. Genome quality (completeness and contamination) was assessed using checkM (Supplementary Table 1) and taxonomy was assigned to each genome using GTDB-Tk v0.3.2 [22].

### Function annotation (ARGs and VFs), plasmid classification and typing, and novel plasmid identification

ARG profiles of the genomes and plasmids were identified by BLASTP against the SARG database at *E* value ≤ 10^-7^ with a minimum similarity of 80% over 70% query coverage [23], and VFs were annotated based on BLASTP homology search (*E* value ≤ 10^-7^) against an experimentally confirmed VF protein database with ≥ 80% identity and coverage [24]. We excluded the analysis of “multidrug type”, a resistance type that usually includes efflux pumps on bacterial membranes that are not necessarily related to ARGs [25]. Plasmid classification and visualization were performed using Plascad [26]. Briefly, plasmids were classified into three categories (conjugative, mobilizable, and non-mobilizable) on the basis of protein machinery associated with DNA transfer, including relaxase, type IV coupling protein, and type IV secretion systems [27]. A plasmid was considered conjugative if it carried relaxase, T4CP, and T4SSs and mobilizable if it encoded only relaxase, whereas plasmids missing all these elements were classified as non-mobilizable. For all the identified conjugative plasmids in our isolates, see Supplementary Fig. 1. All plasmids were searched against the replicon marker sequences using Plasmidfinder [28]. Potential novel plasmids in our isolates were identified using CD-HIT [29] and BLASTn against all publicly available complete plasmids (*n* = 17,906, Aug 2019, Supplementary Table 2); only plasmids with similarity and coverage less than 90% and 95%, respectively, were considered potential novel.

### Pangenome analysis

Pangenome analysis of all the MDR isolates was performed using Anvi’o v5.5 [30]. In brief, we generated a database for each genome along with the gene annotation information using the command “Anvi-gen-contigs-database” with default settings. After creating a storage database with the “Anvi-gen-genomes-storage” command, the “Anvi-pangenome” command was used to compute the pangenome with the following parameters (--use-ncbi-blast, --minbit 0.5, --mcl-inflation 10). The average nucleotide identity (ANI) across all the MDR isolates was calculated using PyANI [31].

To investigate the difference in plasmid composition between MDR isolates, we used Jaccard index defined as the size of the intersection (i.e., shared plasmids between each pair of isolates) divided by the size of the union (i.e., total plasmids in each pair of isolates).

### Phylogeny-based analysis of major MDR isolates and multidrug resistance plasmids

To characterize the phylogenetic relationship between the major WWTP-associated MDR isolates (i.e., *K. pneumoniae* and *E. flexneri*) and relevant species from different sources, we downloaded all publicly available complete bacterial genomes in NCBI (*n* = 15,766, Aug 2019, Supplementary Table 2), and the taxonomy was reassigned using GTDB-Tk v0.3.2 [22] to retrieve the same species as our isolates for core genome SNP-based phylogenetic comparison. It is important to note that *Shigella flexneri* is renamed as *Escherichia flexneri* as proposed in GTDB taxonomy. A total of 623 *E. flexneri* and 388 *K. pneumoniae* genomes, including both our WWTP isolates and the compiled public genomes, were annotated using Prokka [32]. Then, maximum likelihood trees were inferred from the core gene (genes present in > 99% isolates) alignment produced by Roary [33] (aligning with the MAFFT option) using RAxML with a GTR model [34], and the pairwise cSNP distances between genomes were computed using snp-dists (https://github.com/tseemann/snp-dists). Multilocus sequence typing (MLST) analysis for *E. flexneri* and *K. pneumoniae* was performed using mlst (https://github.com/tseemann/mlst) and Kleborate (https://github.com/katholt/Kleborate), respectively. The trees were visualized and annotated (isolation sources and geographic origins) using ITOL v4 [35].

The plasmids carrying ARGs conferring simultaneous resistance to the four selective antibiotics in our WWTP isolates (*n* = 39), as well as those relevant plasmids (*n* = 46) identified in the NCBI plasmid database (*n* = 17,906, Aug 2019, Supplementary Table 2), were analyzed using Roary [33], and the phylogeny and genomic data (e.g., ARGs and conjugative elements) were visualized in Phandango [36].

### Bipartite network construction of plasmids

To investigate the possibility of ARG/ARG cluster transfer between different categories of plasmids, AccNET [37] was used to construct a bipartite network connecting the genomic units and the PCs (protein clusters). The visual location of plasmids in the network was determined using ForceAtlas2 [38] as a layout algorithm, which arranged the plasmids according to the set of shared proteins. Broad-range proteins tend to lie in the center of this network, while narrow-range proteins tend to lie outside of the network. Gephi [39] software was used for network visualization and manipulation.

### Identification of recently transferred ARGs, ISs, and toxin–antitoxin systems

The relative recently transferred ARGs were defined as the identical ARGs (100% nucleotide identity and coverage) present across different genetic backgrounds identified using BLASTN (*E* value ≤ 10^-7^). ISs were characterized based on homology search against the ISfinder database [40] with BLASTN (*E* value ≤ 10^-7^), matches were filtered using thresholds for identity (80%) and coverage (80%), and only those passing the threshold were kept for further analysis. To identify adjacent ISs with the best likelihood of mediating the transfer of ARGs, we limited the search to the 5-kb regions at both ends of the shared ARGs. In this case, the coverage of ISs was not considered, allowing the inclusion of truncated ISs due to their dynamic evolution. However, the arrangement of ARGs and their associated adjacent ISs (i.e., same nucleotide distance between each ARG-IS pair in different genetic backgrounds) was used as an additional filter for the confirmation of potential IS-associated transfer [26]. To identify toxin–antitoxin systems, we used hmmsearch (alignment length > 50% and *c* value < 0.01) against TASmania HMM profiles [41].

### Conjugation assay

To confirm the transferability of the predicted conjugative plasmids, a conjugation assay was performed on 14 representative ARG-bearing conjugative plasmids, which covered all the isolation sources (influent and effluent of the three WWTPs); a wide-sized range (35,925–290,014 kb) and various number of ARGs carried on the corresponding conjugative plasmids (2–19) because our WWTP isolates are resistant to multiple antibiotics, thus limiting the selection of suitable recipients to confirm the transfer of all the predicted conjugative plasmids. We used two different recipients (i.e., tigecycline-resistant *E. coli* DH5α and gentamicin-resistant *E. coli* DH5α) for conjugation experiments based on the phenotype of the conjugative plasmids in the donor bacteria.

Both donor and recipient cells were cultured in LB at 37 °C overnight. One-milliliter donor cells and 200-μL recipient cells were washed three times with NaCl solution (0.85%, w/v), mixed and inoculated onto a 0.45-μm membrane placed on the surface of a LB agar plate. Bacteria on the membrane were resuspended in LB and serial diluted after incubation at 37 °C overnight. The diluted culture was plated on LB agar plates supplemented with the corresponding selective antibiotics (kanamycin: 50 mg/L; tetracycline: 20 mg/L; tigecycline: 10 mg/L; gentamicin: 50 mg/L). The conjugation efficiency was calculated by dividing the number of transconjugants by the number of recipient cells.

## Results

### High prevalence and heterogeneity of MDR bacteria in WWTPs

To examine the prevalence of MDR bacteria in WWTPs and their capacity to exchange ARGs with human/animal-associated pathogens, we isolated MDR bacteria resistant to a combination of four categories of antibiotics (ampicillin, kanamycin, tetracycline, and chloramphenicol) from the influent and effluent of three WWTPs. For each influent and effluent, we randomly selected at least 12 and 15 MDR isolates, respectively (82 altogether). We sequenced all the selected isolates using Illumina and Oxford Nanopore platforms and assembled and analyzed their genomes (including 264 circular plasmids).

**Fig. 1:**
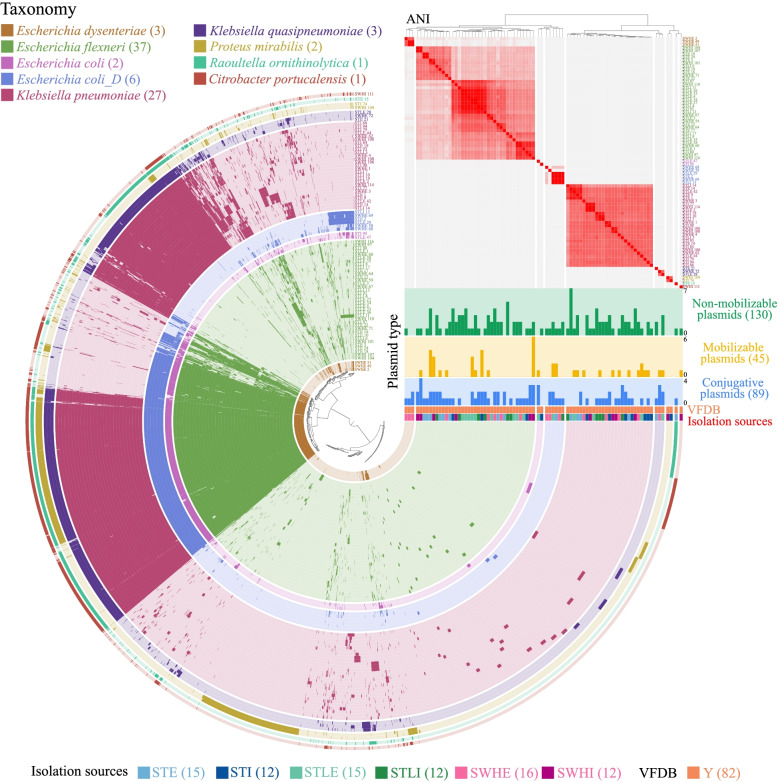
Pangenome analysis of 82 MDR isolates. Each concentric circle represents a bacterial genome, with different colors indicating different bacterial species. Taxonomic classification based on GTDB-Tk v0.3.2. Bars in each layer indicate the occurrence of gene clusters in each genome. Gene clusters are organized based on their distribution across genomes, and genomes are ordered based on ANI (range: 98–100% are shown), as depicted by the red heatmap in the upper right. Plasmid categories (conjugative, mobilizable, and non-mobilizable), virulence factor (Y: present) and isolation sources (influent or effluent from the three WWTPs) are indicated below the heatmap

All the obtained isolates were identified as pathogens (i.e., presence of virulence genes) belonging to five genera (Fig. 1 and Supplementary Table 3). The majority of isolates were classified as *Escherichia flexneri* (equivalent to *Shigella flexneri* in NCBI taxonomy; *n* = 37, 45.1%) and *Klebsiella pneumoniae* (*n* = 27, 32.9%). Both species were prevalent in the influent and effluent of the three WWTPs (Fig. 1 and Supplementary Table 3). We also identified a total of 264 circular plasmids, of which more than half (*n* = 141, 53.4%) were potentially novel plasmids based on similarity search (< 95% similarity and 90% coverage) against the plasmid database. Overall, most isolates (*n* = 65, 79.3%) were predicted to have at least two plasmids. Interestingly, the plasmid composition differed significantly among these MDR isolates (Supplementary Fig. 2).

To better understand the plasmid composition, we classified them into three categories based on the protein machinery associated with DNA transfer (Fig. 1 and Supplementary Table 3): conjugative (*n* = 89), mobilizable (*n* = 45), and non-mobilizable (*n* = 30). Notably, conjugative plasmids were prevalent (*n* = 57, 69.5%) among these isolates, with an average number of 1.6.

### Evolutionary relatedness between WWTP- and human/animal-associated species

**Fig. 2:**
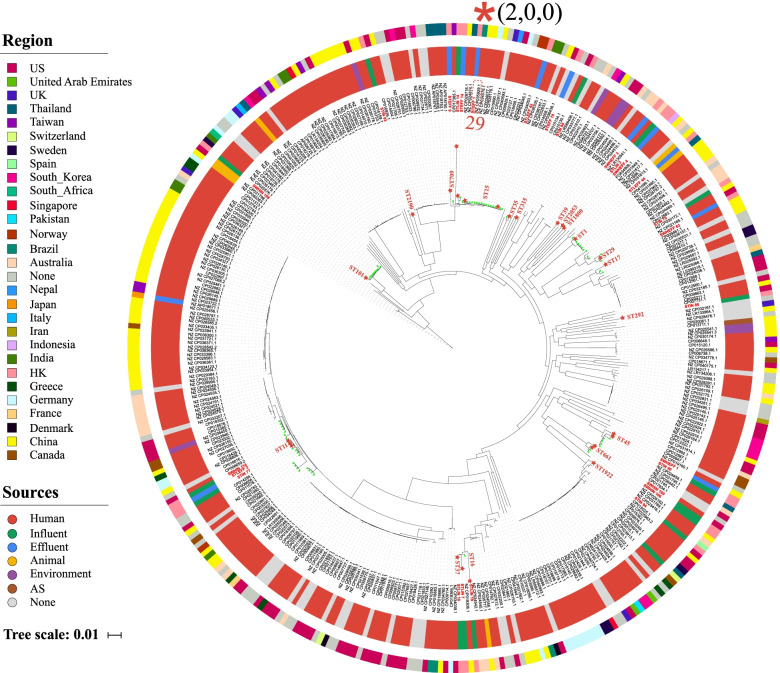
Phylogeny of a total of 388 *Klebsiella pneumoniae* (HK WWTPs: 27; Public: 361) based on core genome SNPs. The inner colored ring indicates the isolation sources, and the outer colored ring indicates the geographical location of isolates. Our WWTP isolates are shown in red, with MLST information included. Green stars adjacent to the STs names indicate global isolates with the same STs as the WWTP isolates. The closely related isolates (defined as differing by < 30 core genome SNPs) between WWTPs and humans (red asterisks) are highlighted, with the adjacent number showing core genome SNPs

To characterize the evolutionary relatedness between WWTP-associated species and those of relevant species from different isolation sources (mainly from humans and animals), we combined core-genome single-nucleotide polymorphism (SNP)–based phylogenetic analysis and plasmid profiling. We found broad phylogenetically distinct clusters featuring 19 different STs for both WWTP-associated *K. pneumoniae* (nine WWTP-specific STs, Fig. 2) and *E. flexneri* (10 WWTP-specific STs, Supplementary Fig. 3). This result was consistent with the observation that isolates within each of the above species varied considerably from each other by a median of 8856 SNPs (range 11–11,260) and 2087 SNPs (range 8–3309), respectively (Supplementary Table 4), suggesting the significance of WWTPs as an important reservoir of highly diverse MDR populations.

Although most WWTP-associated MDR *K. pneumoniae* and *E. flexneri* clustered together with human/animal-associated isolates that were predicted to be pathogens (i.e., encoding experimentally confirmed VFs) (Fig. 2 and Supplementary Fig. 3), pairwise SNP analysis indicated that these WWTP-associated isolates were genetically distinct from their closest human/animal-associated relatives in the public database (defined as > 100 core genome SNPs; avg. 536 and 2501 pairwise SNPs for *E. flexneri* and *K. pneumoniae*, respectively, Supplementary Table 5), and furthermore, we observed substantial differences in the plasmid composition that clearly distinguished each pair (Supplementary Fig. 4). Of the 20 paired comparisons of WWTP-associated *E. flexneri* and their closest human/animal-associated relatives in the public database, only three pairs shared limited plasmids (Avg. Jaccard similarity = 0.31), and no shared plasmids were identified between the 18 paired *K. pneumoniae* comparisons.

By contrast, our phylogenetic analysis indeed identified several linkages with a high degree of relatedness (defined as differing by < 30 core SNPs; avg. 17.25, Supplementary Table 5) between isolates from WWTPs and humans (e.g., *K. pneumoniae* ST15 STEFF1/CP035929.1; *E. flexneri* ST10 STIN87/CP010371.1 and ST44 SWHIN110/CP019005.1) or animals (*E. flexneri* ST744 STLEFF36/CP023383.1 and ST46 STIN80/CP023377.1), indicating that the epidemic potential of WWTP-based resistance transmission may be linked to some particular generalist clones that can colonize different types of hosts. However, we observed substantial differences in the plasmid profiles for the above closely related pairs, and there was almost no overlap in the plasmid composition, although each pair contained at least two plasmids (Supplementary Fig. 4 and Supplementary Table 5). This suggests that different configurations of plasmids may be critical for the adaptation and survival of evolutionarily related populations in different ecological niches. In addition, we observed that although the closely related pair from the same WWTP source shared some plasmids, the overall profile of plasmids in different hosts differed significantly (Supplementary Fig. 4 and Supplementary Table 5). For instance, *E. flexneri* STLEFF36 and STLEFF33 (13 SNPs, average nucleotide identity (ANI) > 99.9%) shared 3 out of a total of 7 plasmids, whereas no plasmid was shared between STLEFF36 and STLEFF4 (each contained 3 plasmids, 27 SNPs, ANI > 99.9%). The high evolutionary dynamics of plasmids within closely related lineages, even within the same ecological niche, may be important for providing bacterial populations with access for rapid capture and turnover of different adaptive traits, such as antibiotic resistance and virulence determinants.

### Plasmids are the main source of multidrug resistance

**Fig. 3:**
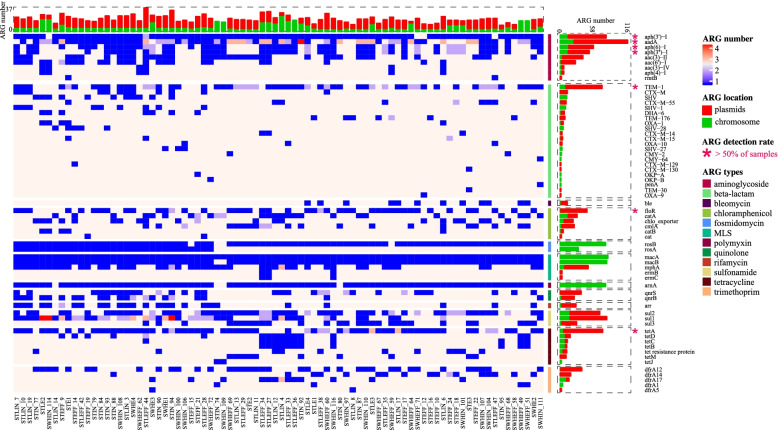
Plasmids play an important role for the horizontal transfer of ARGs. A heatmap is plotted showing the prevalence and distribution of the ARGs identified in the 82 MDR isolates. ARGs present in more than half isolates are highlighted with dark pink asterisks. Red and green bars indicate the number of ARGs carried by plasmids and chromosomes, respectively

To verify the contribution of plasmids to the multidrug resistance phenotype, we examined the ARG profiles for each isolate. This analysis revealed an abundant (*n* = 1569, 9–36 ARGs/cell, avg. 19.1) and diverse collection of ARGs (13 types and 64 subtypes, Fig. 3 and Supplementary Table 6). The majority of the isolates (*n* = 74, 90.2%) were predicted to be resistant to ≥ 9 antimicrobial classes. Given that only four antibiotics were used for the selection, this result demonstrates the high rate of cooccurrence of different resistance categories. Notably, we found that plasmids were the main source of resistance for most isolates, as revealed by the significant difference (Mann–Whitney test, *P* < 0.001) in ARG number between chromosomes (3–25, avg. 7.5) and plasmids (0–30, avg. 11.6). Consistently, the plasmids had a significantly higher number of ARGs for most detected subtypes (*n* = 42, 64.6%) than the chromosomes (avg. 22.0 vs 4.3, Mann–Whitney test, *P* < 0.001), whereas only some ARG subtypes associated with efflux pumps [42, 43], such as *rosA*, *rosB*, *macA*, and *macB* were exclusively carried on chromosomes (Fig. 3 and Supplementary Table 6).

To investigate the association between AMR phenotypes and genotypes, we identified the key genetic determinants conferring resistance to the four selective antibiotics used in this study. By investigating the prevalence of each ARG subtype, we found a total of 15 subtypes in more than half of the isolates. Furthermore, we identified the combination of *aph(3’)-I* (aminoglycoside), *bla*_TEM-1_ (beta-lactam), *floR* (chloramphenicol), and *tetA* (tetracycline) as the major cause of MDR to the selective antibiotics, given their high prevalence and the previous validation of their phenotypes [44–47] (Fig. 3). We then examined the genetic context of these ARGs, as well as those of low abundance but functionally related genes, such as *bla*_CTX-M_, *bla*_OXA_, *catA*, *tetB*, *tetC*, and *tetD*, and confirmed that plasmids were associated with these phenotypes. Accordingly, the MDR phenotype of 62.2% (*n* = 51) of isolates could be explained by the expression of only plasmid-borne ARGs, whereas only 7 isolates were completely dependent on the expression of chromosomal resistance genes (Supplementary Table 6). Interestingly, the cooccurrence of four different types of resistance on a single plasmid was very common (*n* = 46, 56.1%; 52.2% were conjugative); moreover, some ARG subtypes, such as *aadA*, *aph(6)-I*, *aph(3”)-I*, *sul1*, *sul2*, and *mphA* were also surprisingly widespread on these plasmids (Fig. 3). In such cases, the acquisition of single plasmids can mediate the transfer of co-localized ARGs.

### Wide dissemination of WWTP plasmids carrying dynamic ARGs

**Fig. 4.**
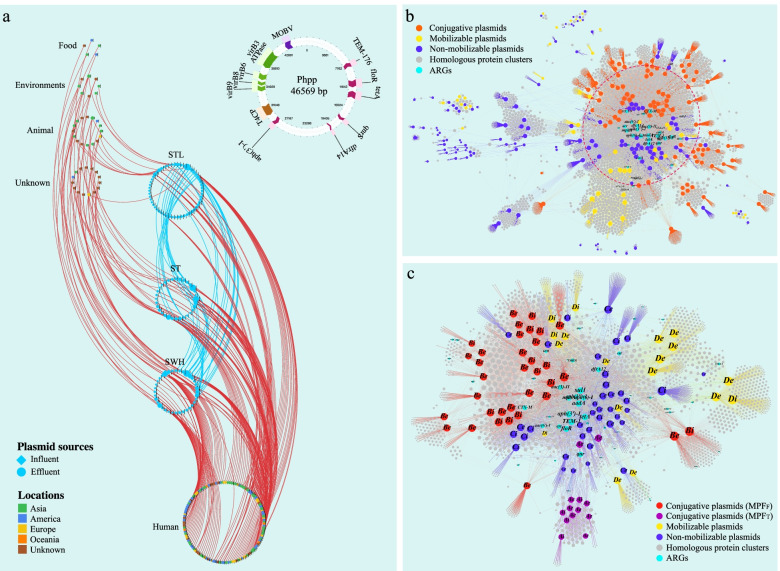
Network diagram depicting the global dissemination of WWTP plasmids. **a** Each node represents a plasmid, with different colors showing geographical locations of the plasmids. The curved lines represent the plasmids that are shared between different isolation sources. A representative MDR conjugative plasmid that may be transferred between different bacterial genus (*Escherichia* and *Salmonella*) across geographical and ecological barriers (i.e., detected in three Hong Kong WWTPs and a wet market in Singapore) is shown in the upper right. **b** Bipartite network of horizontal gene transfer among all plasmids (conjugative: 89; mobilizable: 45; non-mobilizable: 130). The location of the plasmids in this figure is determined using ForceAtlas2 as the layout algorithm by connecting the genomic units (i.e., plasmids) and homologous protein clusters (HpCs). This layout arranges the plasmids by the set of shared proteins among these plasmids and the broad-range determinants tend to lie in the center of the network. The shared ARGs are highlighted in vivid cyan, and different categories of plasmids are indicated. **c** Bipartite network of horizontal gene transfer among all ARG-bearing plasmids (conjugative: 48; mobilizable: 18; non-mobilizable: 38). The node color and the capital letter in each node represents the plasmid type (i.e., A: conjugative MPF_T_; B: conjugative MPF_F_; C: non-mobilizable; D: mobilizable), and the lowercase letter indicates the isolation source of the corresponding plasmid (i.e., i: influent; e: effluent)

Given the high diversity of plasmids and their strong association with ARGs, we examined the potential transfer of the plasmids between the WWTP-associated isolates and other bacteria in different habitats. We compared the plasmid sequences of our isolates with those of a collection of 17,906 complete plasmids and found that a total of 296 plasmids (123 from our WWTP isolates and 173 from the public database), 82 of which carried ARGs, were widely distributed across different habitats (Fig. 4 and Supplementary Table 7). However, although a substantial phylogenetic divergence of the plasmid hosts and a remarkable difference in the overall plasmid profiles was observed, most potential transfer (59.1%), including 72% of ARG-associated plasmids, occurred within the same bacterial genus or species (Supplementary Table 7). By contrast, our analysis also showed that 40.9% of the plasmids may be able to transfer across bacterial genus barriers, and importantly, all the ARG-bearing plasmids among them were conjugative (Supplementary Table 7).

We found that multiple ARGs were frequently colocalized on single widely disseminated conjugative plasmids. For example, one conjugative plasmid (Phpp, Fig. 4a) carrying six ARGs (*bla*_TEM-176_, *aph*(*3’*)*-I*, *floR*, *tetA*, *qnrS* and *dfrA14*) was present in three phylogenetically distant *Escherichia* isolates (avg. SNPs 39,786, range 25,292–47,405) derived from the effluent of the three WWTPs, and this plasmid was highly similar (> 99% nucleotide sequence identity) to psg_ww281, a plasmid from a *Salmonella enterica* strain isolated from a wet market in Singapore (GenBank accession: NZ_CP037995.1) (Supplementary Table 7). This provides compelling evidence for the significance of conjugative plasmids in facilitating the horizontal transfer of ARGs. We further investigated the transferability of the predicted conjugative plasmids by conjugational transfer of 14 representative ARG-bearing conjugative plasmids (size range: 35,925–290,014 kb; number of ARGs: 2–19) and observed successful transfer of all these plasmids with conjugation efficiency ranged from 8.10 × 10^-7^ to 9.60 × 10^-4^ (Supplementary Table 8).

### Potential transfer of ARGs and ARG clusters across diverse genetic backgrounds

With detailed examination of the shared plasmids observed above, we identified a dynamic variation in the ARG composition (ARG acquisition and loss), which stands in sharp contrast to the high conservation across the rest of the plasmid sequences (Supplementary Table 7). This suggests that the ARGs in plasmids may be in a constant state of flux and can evolve and transfer separately from the plasmids. This prompted us to investigate the possibility of ARG transfer between different categories of plasmids, as well as the major contributors involved in these processes. We first constructed a bipartite network connecting the homologous protein clusters (HPCs) and all WWTP plasmids, which arranged the plasmids according to the set of shared HPCs (Fig. 4b). This network showed a large hub of strongly connected ARG-bearing conjugative plasmids (*n* = 48) and some mobilizable (*n* = 18) and non-mobilizable plasmids (*n* = 38), while many non-mobilizable plasmids without ARGs were sparsely connected to this hub. This observation was also supported by the finding that ARGs were significantly more prevalent among conjugative plasmids than non-mobilizable plasmids (53.9% vs 28.8%, *P* < 0.001).

Careful inspection of this hub revealed potential horizontal transfer of most detected ARGs between different plasmid categories. We found that 77.1% of all ARG subtypes were exchanged between at least two plasmid categories, of which 94.6% showed the involvement of conjugative plasmids. Further investigation revealed that the main conjugative plasmid types included MPF_F_ and MPF_T_ (Fig. 4c), which were widely distributed in both the influent and effluent of the WWTPs.

**Fig. 5:**
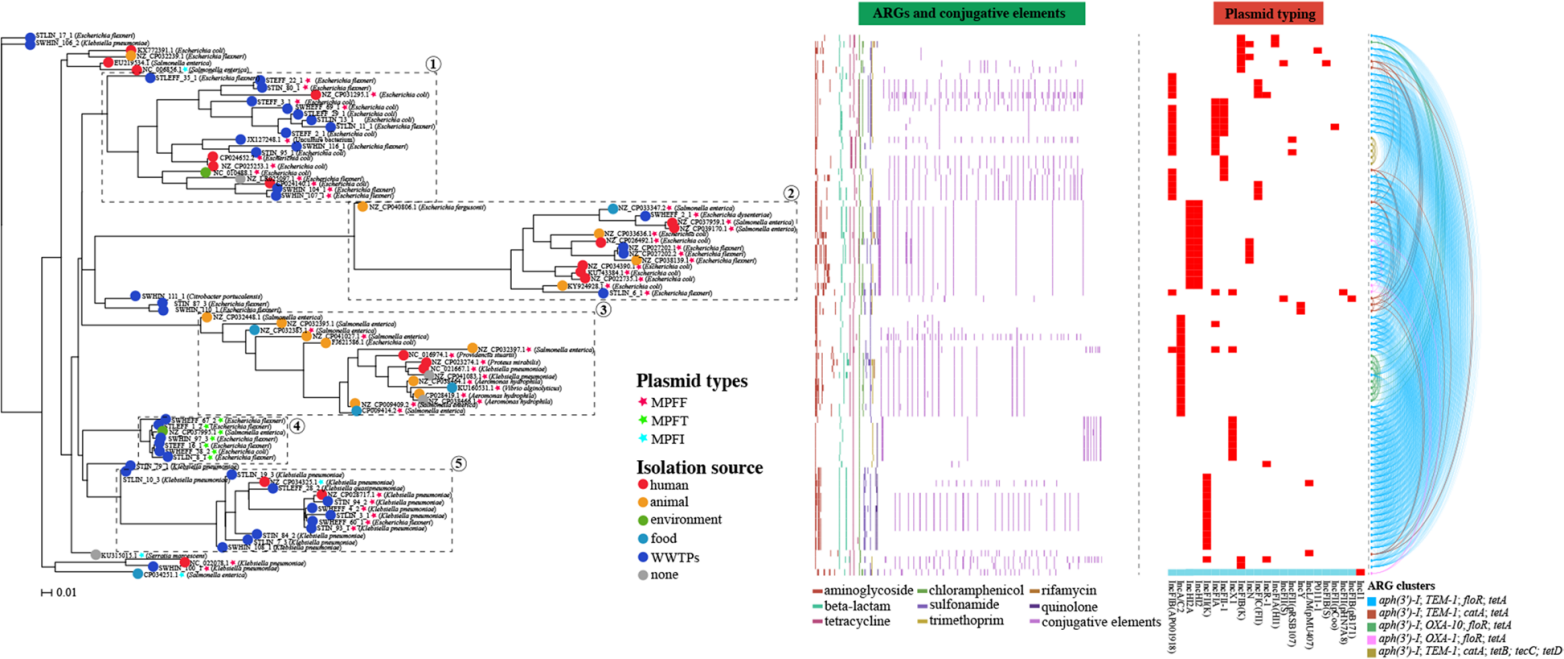
Horizontal transfer of ARG clusters conferring resistance to four antibiotics across diverse plasmid backgrounds (85 in total) and ecological barriers. Hierarchical clustering of plasmids (WWTPs: 39; public: 46) is performed using the binary presence and absence of accessory genes. Colored nodes indicate the isolation source of the plasmids. The conjugative plasmids are depicted by different colored asterisks, as per the inset color key. The presence of ARGs, conjugative elements, and plasmid replicon genes are plotted as a binary heatmap, and different ARG clusters identified across the plasmid data set are shown to the right as the colored nodes, with lines indicating the potential transfer of the corresponding ARG clusters

We expanded our analysis to the potential transfer of ARG clusters between plasmid categories and focused on those conferring simultaneous resistance to the four selective antibiotics. We then carried out a whole genome analysis on all the relevant plasmids from both the WWTP plasmids (*n* = 39) and the publicly available plasmid database (a total of 46 plasmids meets our requirement) to infer the transmission dynamics (Fig. 5). Our data clearly revealed five major distinct plasmid groups (69.1% of plasmids were conjugative) with hosts from nine different genera involved in the potential transfer of a total of five different ARG clusters, which was consistent with the bipartite network analysis in Supplementary Fig. 5a. The majority of the lineages encompassed plasmids obtained from both WWTPs and other sources (mainly human-related pathogens), with the exception of group 3, where the plasmids were derived mainly from animals. Mapping plasmid replicons (23 types) and mobilization protein profile data onto the phylogeny showed a close association with each clustered plasmid group (Fig. 5). We found that each group was associated mainly with a single specific replicon type (IncHI2A/HI2, IncA/C2, IncX1, and IncFII(K) for groups 2–5, respectively), apart from group 1, in which nearly all plasmids contained at least two different replicon types (e.g., IncFIB, IncFIA, and IncFII-1). The substantial heterogeneity of the plasmids in different groups contrasted with the conserved ARG clusters found among them, suggesting that the ARG clusters may be transferred between these plasmids *via* horizontal gene transfer (HGT). Notably, some ARG clusters exhibited extensive dissemination between different plasmids from WWTP- and human-associated bacteria (Supplementary Fig. 5b). For example, a cluster (*bla*_TEM-1_, *aph(3’)-I*, *floR*, and *tetA*) was present among all five plasmid groups with isolation sources from at least 15 different regions, indicating that these ARGs are likely under strong selective pressure in these ecological settings. In addition, many conjugative plasmids were also found to share a variety of ARGs other than the ARG clusters we focused on, such as the *mcr-1* gene in cluster 2 and the *bla*_NDM-1_ gene in cluster 3 (Supplementary Fig. 5a). Collectively, these findings reveal potential transfer of ARGs and ARG clusters between different plasmids across phylogenetic and ecological barriers and highlight the critical involvement of conjugative plasmids in these processes.

To investigate whether the ARG clusters shared by plasmids have the potential to be transferred between the WWTP- and human/animal-associated bacterial chromosomes, we then examined the ARG clusters on the bacterial chromosomes from WWTPs and explored their distribution in the public bacterial database (Supplementary Fig. 6). Importantly, we found that some ARG clusters, such as the cluster (*bla*_TEM-1_, *aph*(*3’*)*-I*, *catA* and *tetBCD*) and the cluster (*bla*_TEM-1_, *aph*(*3’*)*-I*, *catA and tetA*) that were present in the chromosomes of three and five different bacterial genera, respectively, were also shared by different plasmids (Fig. 5 and Supplementary Fig. 5). This result indicates that plasmids may play an important role in mediating the transfer of ARG clusters even between bacterial chromosomes.

### ISs play an important role in mediating the transfer range of ARGs

**Fig. 6:**
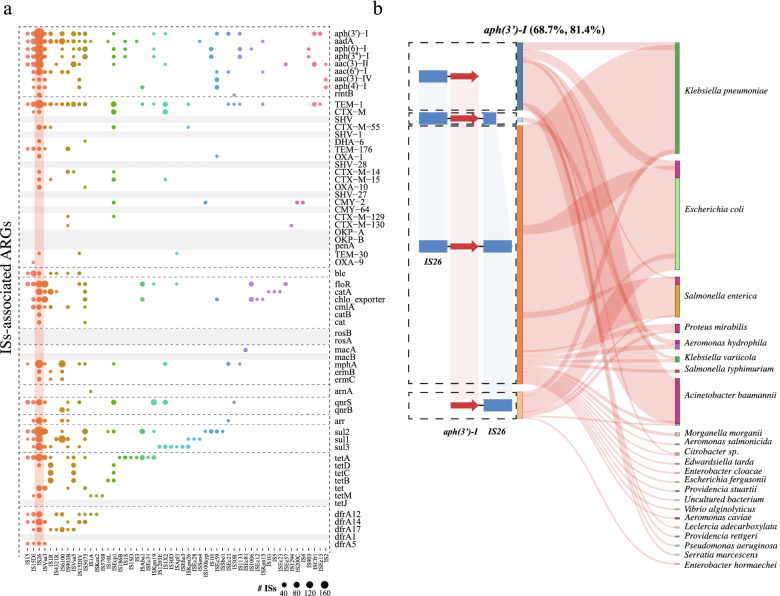
ISs play a critical role in expanding the transfer range of ARGs. **a** A bubble chart is plotted showing the association of ARGs with the adjacent ISs (5 kb upstream and downstream of the ARGs) in the 82 MDR isolates. IS26, which is closely associated with a diverse ARGs, is highlighted with an orange strip. **b** Evidence for IS-associated ARG transfer across different genetic contexts between WWTPs and public datasets. Plasmid-borne aminoglycoside resistance gene (i.e., *aph*(*3’*)*-I*) is shown for visualization purpose. For other ARG subtypes and the chromosomal-borne ARGs, see Supplementary Fig. 7 and Supplementary Table 9. Pairwise synteny maps are plotted displaying the relatively recent transfer patterns (100% nucleic acid identity) of IS-associated ARGs, with the red arrows and blue rectangles indicating the shared ARGs and ISs, respectively. The Sankey diagram displays the plasmid hosts and the chromosomes in the public datasets that are involved in the potential transfer, which matches the IS-ARG sharing patterns on the left. For each bacterial species, red violet bar indicates the proportion of the involved chromosomes, while the rest of each bar indicates the hosts of the plasmids. The two numbers next to each ARG subtype represent the percentage of the ARGs in our WWTP plasmids that are shared with the public datasets and the proportion of IS-associated transfer to total ARG transfer events between WWTPs and other environments (mainly humans/animals), respectively

The potential transfer of ARGs across diverse genetic backgrounds raises the question of what other genetic elements are needed beyond plasmids for the dynamic exchange of ARGs. Narrowing the analysis to the regions adjacent to the ARGs (5 kb upstream and downstream), we found that approximately 80% (*n* = 51) of the ARG subtypes had at least one type of IS nearby (avg. 6.7, range 1–22) (Fig. 6a). A total of 53 IS types were detected, 11 of which were related to more than 10 ARG subtypes. The most noteworthy example was the prevalent association with IS26, which was detected in the adjacent regions of most ARG subtypes (*n* = 42, 65.6%, Fig. 6a). We subsequently focused on the ARGs (i.e., *aph*(*3’*)*-I*, *bla*_TEM-1_, *tetA*, *floR, bla*_OXA-1_, *catA*, *tetB*, *tetC*, and *tetD*) conferring resistance to the selective antibiotics and discovered that they were associated with extremely diverse ISs (Fig. 6a). This suggests the potential role of ISs in generating a dynamic pool of ARGs, which may serve as the principal available source for large-scale exchange across different genetic backgrounds.

To test the above hypothesis, we first explored the relationship of ISs to the relatively recent transfer of plasmid-borne ARGs in the WWTPs by comparing the ARG sequences with those in public databases (both plasmids and chromosomes) based on perfect nucleic acid sequence matches (100% similarity and coverage) and then examined the flanking regions (5 kb upstream and downstream) of these recently transferred ARGs. We demonstrated that most of the potential transfers were tightly linked to the presence of ISs (avg. > 80%; range 65.4–95.6% for *tetB* and *floR*, respectively), as evidenced by the well-conserved pattern in genetic organization and synteny of not only the ARGs but also the flanking ISs (Fig. 6b, Supplementary Fig. 7 and Supplementary Table 9). Notably, the association with ISs has considerably expanded the transfer range of ARGs. For example, IS-associated ARGs, such as *aph*(*3’*)*-I*, *bla*_TEM-1_, *tetA*, and *floR*, were detected in at least 22, 35, 34, and 14 different bacterial species (plasmids or chromosomes), respectively, which was far beyond the host range of the plasmids carrying them (Fig. 6 and Supplementary Fig. 7). In addition, the horizontally transferred ARGs across different bacterial chromosomes were also closely associated with the flanking ISs (Supplementary Table 9). Taken together, our findings support the notion that plasmids (especially conjugative plasmids) in cooperation with ISs [48, 49] shape a multilayered, hierarchical process that can potentially mediate the transfer of ARGs between WWTP- and humans/animal-associated bacteria, and the basic transfer unit (i.e., ISs-ARGs) should be considered as an important indicator for resistance transfer risk assessment.

### Convergent multidrug resistance–hypervirulence plasmids

While ARG-bearing plasmids in WWTPs are of concern, those carrying hypervirulence determinants pose the greatest health threat. We explored the genetic diversity, distribution, and mobilization of plasmid-borne VFs (Supplementary Fig. 8 and Supplementary Table 10). Notably, we identified a total of 27 plasmids (24 were potential novel) carrying a variety of VFs (11 different loci), which were carried mainly by *Escherichia* (77.8%, *n* = 21) and *Klebsiella* (22.2%, *n* = 6). Gene clusters encoding the biosynthesis of the siderophores aerobactin (*iutA*, *iucD*, *iucC*, *iucB*, and *iucA*; *n* = 15) and salmochelin (*iroBCDEN*; *n* = 6), which serve as important biomarkers for hypervirulence [50], were prevalent in these plasmids. Interestingly, these two clusters that are typically colocalized on virulence plasmids of *K. pneumoniae* [50] were commonly detected in plasmids in our *E. flexneri* collection (Supplementary Fig. 8 and Supplementary Table 10). In addition, all these hypervirulence plasmids were conjugative, and their hosts were widely distributed across different STs (Supplementary Table 10)—clearly indicating their widespread presence across different ecological contexts. Of greater concern was the finding that most of the virulence plasmids (59.3%, *n* = 16) also carried multiple ARGs (avg., 10.9; range 2–16), raising the prospect of cotransfer of these phenotypes between WWTP- and humans/animals-associated bacteria (Supplementary Fig. 8). For example, the convergent multidrug resistance–hypervirulence plasmid STEFF_18_plasmid_1 (*iuc* loci + 7 ARGs) was nearly identical (> 99% nucleotide sequence identity) to a plasmid (GenBank accession: CP024827.1) isolated from a human source in South Korea.

Inspection of the genetic contexts surrounding the major VFs revealed that they were closely associated with various ISs across different plasmid backgrounds, with the most common association occurring with IS1. For example, most of the aerobactin operons (87.5%) were closely associated with one or two copies of an IS1 element, and *iroBCDEN* was usually flanked by a copy of IS1 and ISKpn28, alongside an IS2. Two of three type 3 fimbriae-encoding clusters (*mrkABCDF*) were complete and tightly linked to the presence of both IS3 and IS1, while a third cluster lost the *mrkF* gene *via* IS1-mediated transposition (Supplementary Fig. 8). These results indicate that HGT of VFs *via* ISs between different plasmid backgrounds may also be common and, importantly, the combination of hypervirulence determinants and multidrug resistance on conjugative plasmids in WWTPs is a significant potential threat that warrants further attention.

## Discussion

In this study, we investigated the prevalence, genetic diversity, ARGs, and VFs of MDR isolates in WWTPs and investigated the key factors in mediating the potential transfer of ARGs between WWTP- and human/animal-associated bacteria. We showed the importance of generating complete genomes using the combination of Nanopore and Illumina sequencing, which enabled us not only to reconstruct the genomic structure but also to identify the associated plasmids, facilitating the study of mosaic ARGs, virulence regions, and dynamic evolution. The collection of the complete MDR bacterial genomes in this study has expended the number of complete genomes of bacteria isolated from WWTPs in the NCBI database (< 35 as of the time we downloaded the data, Supplementary Table 2), which provide an important reference for studying the dissemination of resistance.

Importantly, we found that the majority of WWTP-associated MDR isolates (i.e., *E. flexneri* and *K. pneumoniae*) were distributed throughout the broad phylogeny that includes a large number of the same species in the database that were isolated from humans/animals globally. Some WWTP isolates formed a monophyletic lineage, different from all other isolates in the public database, plus the 141 potential novel plasmids identified in total, indicating the high diversity and genetic plasticity of MDR bacteria in WWTPs. Interestingly, most of the WWTP-associated isolates were genetically distinct from their closest relatives from humans/animals based on core genome SNPs and plasmid profile analyses, reflecting potential mutually exclusive adaptation strategies. However, we may have underestimated the degree of the overlap between WWTP- and human/animal-associated isolates due to the limited ability of our samples to capture the full spectrum of genetic diversity for complete comparison of these two compartments. In contrast, we identified some lineages that contained isolates with remarkable genome similarity from mixed sources. For example, STIN87 and STLEFF36 were only 10 core SNPs away from a human isolate from Colombia in 2012 (GenBank accession: CP010371.1) and a canine isolate from the UK in 2002 (GenBank accession: CP023383.1), respectively, supporting a high degree of relatedness. Nevertheless, there was no overlap among the composition of plasmids within each of the above pairs; moreover, even closely related isolates from the same WWTPs source can be highly heterogeneous in terms of plasmid profiles, suggesting that the diversification of plasmids may have driven the evolution of closely related populations [51] to develop specific counter strategies in order to survive under different selective conditions. A representative example was the acquisition of adaptive traits exemplified by ARGs; for instance, some variants of the New Delhi metallo-beta-lactamase (*bla*_NDM-4_ and *bla*_NDM-6_) and class C beta-lactamase (*bla*_CMY-2_) were detected in the plasmids of the above human and canine isolates, respectively, but not in their closely related WWTP isolates. These results support the notions that comparative genomic analyses should not focus solely on SNP-based comparisons and that high-resolution profiling of MGEs (e.g., plasmids and ISs) is indispensable for inferring the transmission of resistance, especially considering that plasmids are the main carriers of resistance. Additionally, the degree of selective pressure exerted by antimicrobials should also be considered. It is important to remember that we may have overlooked some forms of ARGs belonging to “multidrug types” (e.g., intrinsic membrane transporters), which may also be related to resistance.

Although the carriage of ARGs on plasmids allows their hosts to rapidly adapt to fluctuating selective conditions, plasmids generally impose a fitness burden owing to the metabolic load of plasmid-borne genes [52]. Investigation of the toxin–antitoxin systems that promote plasmid maintenance [53, 54] revealed that they were significantly enriched on plasmids carrying ARGs (Fisher’s exact test, *P* < 0.01, odds ratio (OR) > 1, Supplementary Fig. 9) and were more prevalent on conjugative plasmids. Interestingly, we found that the coexistence of multiple plasmids in the same MDR strain was very common, which may promote plasmid survival in bacterial populations, as demonstrated by the previous finding that positive epistasis between coinfecting plasmids can minimize the cost of plasmid carriage and increase plasmid stability [55]. Coupling the WWTP plasmids with all the available plasmids in public databases revealed potential transfer of ARG-bearing plasmids between WWTP- and humans/animal-associated bacteria. However, it is difficult to account for the ability of bacteria to spread ARGs by the transfer of plasmids alone because most transfers happened within the same bacterial species or genus, even for conjugative plasmids that were able to transfer across genera, for which the transfer range was limited to the *Enterobacteriaceae* family (Supplementary Table 7). Occurrence was much less than that of the transferred ARGs detected within the same database (across family level), implying the potential barrier for ARG transfer mediated by plasmids alone [56]. This finding is reasonable since shared evolutionary history of bacteria is usually associated with the overlap in the host range of mobile elements [57].

Notably, we observed potential horizontal transfer of ARGs and ARG clusters across distinct bacterial genetic backgrounds between WWTPs and human/animal sources, indicating the dynamic transfer of ARGs not only *via* plasmids. Detailed analysis of the flanking regions of recently transferred ARGs demonstrated the close association between ARGs and ISs, and the most noteworthy was the dominant contribution of the IS6 family to the transfer of antibiotic resistance. Additionally, although we found that ARGs are mainly carried by plasmids, ICEs may also play a role in the transfer of other resistance not analyzed in this study [58]. Our results highlight the importance of long-read surveillance to capture the key transfer units (i.e., ISs-ARGs) and track their fate across different genetic and ecological boundaries. We found that ISs may also have played an important role in plasmid evolution by maintaining the plasticity required to balance the cost of plasmid maintenance. For example, some multidrug resistance plasmids eliminated the segments encoding the conjugal transfer and toxin–antitoxin and plasmid SOS inhibition systems that may place a fitness burden on their hosts [59] (Supplementary Fig. 10), which was in sharp contrast to the highly conserved multidrug resistance clusters.

WWTPs greatly decreased the MDR bacterial load from (6.27 ± 0.121) × 10^5^/L–(1.38 ± 0.037) × 10^6^/L in the influent to (5.70 ± 0.794) × 10^2^/L–(2.49 ± 0.159) × 10^3^/L in the effluent in this study; however, it is difficult to predict the fate of MDR pathogens that are released into the receiving environments, as a complex array of evolutionary, ecological, and environmental factors would affect the community structure [1]. Additionally, the cooperation between conjugative plasmids and ISs has the potential to produce yet more spread of multidrug resistance into new species and new genomic backgrounds.

Our study is subject to several important limitations. First, the selective medium and the antimicrobials selected for resistance would provide a limited perspective of the antibiotic resistant bacteria and plasmids within WWTPs; however, the approach and workflow described in this study will be an important step forward in elucidating the potential transmission of ARGs between WWTP- and human/animal-associated bacteria. Another limitation is the dependence on the analysis of the public plasmid and genome sequence databases, which are biased toward pathogen species. Therefore, we were likely to underestimate the possible transfer of plasmids and ARGs in other environmental bacteria. Routine surveillances of a wider pool of environmental species and samples are important if we wish to capture a comprehensive picture of the dynamic transfer. Lastly, direct evidence of plasmid/IS-associated ARG transfer between WWTP- and human/animal-associated strains, as well as the determination of the direction of transmission, requires further research on the premise of combining whole genome sequencing and well-collected epidemiological data.

## Conclusions

In this work, we used population genetics to analyze complete genomes of MDR isolates from WWTPs, establishing high-resolution epidemiological evidence of resistance gene transfer between WWTP- and human/animal-associated bacteria. Our study showed that potential transfer of multidrug resistance between WWTP- and human/animal-associated bacteria was linked through a multilayered hierarchical process in which the synergy between plasmids (especially conjugative plasmids) and ISs played a key role.

## Supplementary Information

**Additional file 1:** **Supplementary Table 1.** Quality evaluation of the assembled MDR isolates (82 in total).

**Additional file 2:** **Supplementary Table 2.** Summary of the publicly available complete bacterial plasmids and genomes used in this study.

**Additional file 3:** **Supplementary Table 3.** Summary of the assembled complete MDR isolates with Nanopore and Illumina reads (82 in total).

**Additional file 4: Supplementary Table 4.** Summary of pairwise core genome SNPs for *Escherichia flexneri* (623 in total) and *Klebsiella pneumoniae* (388 in total).

**Additional file 5: Supplementary Table 5.** Summary of the most closely related isolates to WWTP *Escherichia flexneri* and *Klebsiella pneumoniae* in public NCBI datasets.

**Additional file 6:** **Supplementary Table 6.** Summary of all the detected ARGs.

**Additional file 7:** **Supplementary Table 7.** Wide dissemination of WWTP plasmids carrying dynamic ARGs.

**Additional file 8:** **Supplementary Table 9.** Summary of recent IS-associated ARG transfer between WWTPs and public datasets.

**Additional file 9:** **Supplementary Table 10.** Summary of all the identified virulence plasmids (27 in total).

**Additional file 10: Supplementary Figure 1.** The schematic view of the genetic constitution of all the identified conjugative plasmids using Plascad. **Supplementary Figure 2.** Differences in plasmid composition among WWTPs-associated MDR isolates. a, Pairwise similarity of plasmid composition (measured by Jaccard index). b, Jaccard index frequency distribution (including all Jaccard similarity: 0-1). c, Zoom view of Jaccard index (Jaccard similarity above 0) frequency distribution. **Supplementary Figure 3.** Phylogeny of a total of 623 *Escherichia flexneri* (WWTPs: 37; Public: 586) based on core genome SNPs. **Supplementary Figure 4.** Distinct plasmid profiles between WWTPs-associated isolates (a: *E. flexneri*; b: *K. pneumoniae*) and their closet human/anima-associated relatives in the public database. **Supplementary Figure 5**, a, Bipartite network of potential horizontal transfer of ARGs among plasmids. Five major plasmid clusters are indicated by white dotted circles with cluster numbers (1-5), which match the five lineages identified in Fig. 5. The most widely transferred ARG cluster (i.e., *aph(3’)-I*, *bla*_TEM-1_, *floR*, and *tetA*) is highlighted by vivid cyan dotted circle in the center of the network. b, Global distribution of the ARG clusters. The size of each node indicates the number of plasmids carrying the corresponding ARG clusters, and the colored lines denote the corresponding clusters. Pie charts are plotted showing the summary of all the plasmids involved. **Supplementary Figure 6.** Potential horizontal transfer of ARG clusters conferring resistance to four antibiotics across diverse chromosome backgrounds (25 in total) and ecological barriers**.** a, Global spread of the ARG clusters. The size of each node indicates the number of bacterial chromosomes carrying the corresponding ARG clusters, and the colored lines denote the corresponding clusters. b, Maximum likelihood phylogenetic analysis of a total of 25 genomes (WWTPs: 8; Public: 27) carrying the shared ARGs clusters. The colored names denote the corresponding ARG clusters. **Supplementary Figure 7.** ISs play a critical role in expanding the transfer range of ARGs. **Supplementary Figure 8.** VFs are closely associated with ISs in plasmids. a, Genetic maps for the co-localized hypervirulence determinants (*iuc* and *iro* loci). b, Plasmids with only *iuc* loci. c, Plasmids with *mrk* loci. Genes that make up all the VFs are highlighted with red arrows. ISs and ARGs are indicated (IS1, IS2, ISKpn28, IS3, and IS26 are specified and other ISs are colored green). MDR-hypervirulent plasmids are highlighted with dark pink asterisk on the left. Grey shading indicates homology blocks sharing between different plasmids. **Supplementary Figure 9.** Mosaic plots showing the relationship between toxin-antitoxin systems, ARGs and categories of plasmids by Fisher’s exact test. **Supplementary Figure 10.** ISs play an important role for plasmid evolution by maintaining the plasticity necessary to balance the cost of plasmid maintenance.

## Data Availability

All the assemblies were deposited into the NCBI SRA database with the following accession number: PRJNA603241.

## Abbreviations

WWTPs: Wastewater treatment plants
MLST: Multilocus sequence typing
MDR: Multidrug resistance
ARGs: Antibiotic resistance genes
VFs: Virulence factors
ISs: Insertion sequences
MGEs: Mobile genetic elements
HPCs: Homologous protein clusters
HGT: Horizontal gene transfer
