# Supplementary material for: High-resolution genomic surveillance elucidates a multilayered hierarchical transfer of resistance between WWTP- and human/animal-associated bacteria
Source: Microbiome. 2022 Jan 25;10:16. doi: 10.1186/s40168-021-01192-w (PMC8790882; doi:10.1186/s40168-021-01192-w)
Supplement: Supplementary file 11 — Additional file 10: Supplementary Figure 1. The schematic view of the genetic constitution of all the identified conjugative plasmids using Plascad. Supplementary Figure 2. Differences in plasmid composition among WWTPs-associated MDR isolates. a, Pairwise similarity of plasmid composition (measured by Jaccard index). b, Jaccard index frequency distribution (including all Jaccard similarity: 0-1). c, Zoom view of Jaccard index (Jaccard similarity above 0) frequency distribution. Supplementary Figure 3. Phylogeny of a total of 623 Escherichia flexneri (WWTPs: 37; Public: 586) based on core genome SNPs. Supplementary Figure 4. Distinct plasmid profiles between WWTPs-associated isolates (a: E. flexneri; b: K. pneumoniae) and their closet human/anima-associated relatives in the public database. Supplementary Figure 5, a, Bipartite network of potential horizontal transfer of ARGs among plasmids. Five major plasmid clusters are indicated by white dotted circles with cluster numbers (1-5), which match the five lineages identified in Fig. 5. The most widely transferred ARG cluster (i.e., aph(3’)-I, blaTEM-1, floR, and tetA) is highlighted by vivid cyan dotted circle in the center of the network. b, Global distribution of the ARG clusters. The size of each node indicates the number of plasmids carrying the corresponding ARG clusters, and the colored lines denote the corresponding clusters. Pie charts are plotted showing the summary of all the plasmids involved. Supplementary Figure 6. Potential horizontal transfer of ARG clusters conferring resistance to four antibiotics across diverse chromosome backgrounds (25 in total) and ecological barriers. a, Global spread of the ARG clusters. The size of each node indicates the number of bacterial chromosomes carrying the corresponding ARG clusters, and the colored lines denote the corresponding clusters. b, Maximum likelihood phylogenetic analysis of a total of 25 genomes (WWTPs: 8; Public: 27) carrying the shared ARGs clusters. The [file 40168_2021_1192_MOESM10_ESM.docx]

**Supplementary information**

**High-resolution genomic surveillance elucidates a multilayered hierarchical transfer of resistance between WWTPs and human/animal-associated bacteria**

You Che^1,3^, Xiaoqing Xu^1^, Yu Yang^1^, Karel Břinda^2,3^, William Hanage^3^, Chao Yang^4^*, Tong Zhang^1^*

^1^Environmental Microbiome Engineering and Biotechnology Laboratory, Center for Environmental Engineering Research, Department of Civil Engineering, The University of Hong Kong, Hong Kong.

^2^Department of Biomedical Informatics, Harvard Medical School, Boston, USA

^3^Center for Communicable Disease Dynamics, Department of Epidemiology, Harvard T. H. Chan School of Public Health, Harvard University, Boston, Massachusetts, USA

^4^Key Laboratory of Molecular Microbiology and Technology for Ministry of Education, Nankai University, Tianjin, 300071, China

**Supplementary Tables**

**Supplementary Table 1 – Quality evaluation of the assembled MDR isolates (82 in total).**

Provided in a separate Excel spreadsheet.

**Supplementary Table 2 – Summary of the publicly available complete bacterial plasmids and genomes used in this study.**

Provided in a separate Excel spreadsheet.

**Supplementary Table 3 - Summary of the assembled complete MDR isolates with Nanopore and Illumina reads (82 in total).**

Provided in a separate Excel spreadsheet.

**Supplementary Table 4 – Summary of pairwise core genome SNPs for *Escherichia flexneri* (623 in total) and *Klebsiella pneumoniae* (388 in total).**

Provided in a separate Excel spreadsheet.

**Supplementary Table 5 - Summary of the most closely related isolates to WWTP *Escherichia flexneri* and *Klebsiella pneumoniae* in public NCBI datasets.**

Provided in a separate Excel spreadsheet.

**Supplementary Table 6 – Summary of all the detected ARGs.**

Provided in a separate Excel spreadsheet.

**Supplementary Table 7 – Wide dissemination of WWTP plasmids carrying dynamic ARGs.**

Provided in a separate Excel spreadsheet.

**Supplementary Table 8 –Conjugation frequency for the selected plasmids.**

**Supplementary Table 9 – Summary of recent IS-associated ARG transfer between WWTPs and public datasets.**

Provided in a separate Excel spreadsheet.

**Supplementary Table 10 - Summary of all the identified virulence plasmids (27 in total).**

Provided in a separate Excel spreadsheet.

Table 8. Conjugation frequency for the selected plasmids.

| **Donor** | **Target plasmid genotype^a^** | **# ARGs** | **Size (bp)** | **Donor selective phenotype^b^** | **Recipient phenotype** | **Transconjugants phenotype** | **Conjugation frequency^c^** |
| --- | --- | --- | --- | --- | --- | --- | --- |
| STEFF_17 | *qnrS*; *aph(3')-I* | 2 | 35,925 | Kan^R^, Tgc^S^ | Kan^S^, Tgc^R^ | Kan^R^, Tgc^R^ | 9.76 × 10^-5^ |
| STIN_94 | *sul1*; *aadA*; *arr*; *aac(6')-I*; *aph(3')-I*; *aac(3)-II*; *sul2*; *aph(3'')-I*; *aph(6)-I*; *mphA*; *tetA*; *floR*; *TEM-1*; *CTX-M*; *qnrS* | 15 | 137,932 | Kan^R^, Tgc^S^ | Kan^S^, Tgc^R^ | Kan^R^, Tgc^R^ | 1.14 × 10^-5^ |
| STLIN_6 | *tetA*; *sul1*; *arr*; *catB*; *OXA-1*; *aac(6')-I*; *aac(3)-IV*; *aph(4)-I*; *sul2*; *ble*; *sul1*; *aadA*; *dfrA12*; *aph(3')-I*; *sul3*; *aadA*; *cmlA*; *aadA*; chloramphenicol exporter | 19 | 290,014 | Kan^R^, Tgc^S^ | Kan^S^, Tgc^R^ | Kan^R^, Tgc^R^ | 7.62 × 10^-6^ |
| SWHEFF_59 | *TEM-176*; *aadA*; *aph(3')-I*; *sul3*; *floR* | 5 | 47,227 | Kan^R^, Tgc^S^ | Kan^S^, Tgc^R^ | Kan^R^, Tgc^R^ | 2.14 × 10^-5^ |
| SWHEFF_60 | *sul2*; *aph(3'')-I*; *aph(6)-I*; *aph(3')-I*; *aac(3)-II*; *aac(6')-I*; *arr*; *aadA*; *sul1*; *qnrB*; *sul1*; *mphA*; *tetA*; *floR*; *TEM-1*; *CTX-M*; qnrS | 17 | 140,589 | Kan^R^, Tgc^S^ | Kan^S^, Tgc^R^ | Kan^R^, Tgc^R^ | 1.52 × 10^-5^ |
| SWHIN_97 | *qnrS*; *aph(3')-I*; *dfrA14*; *floR*; *tetA*; *TEM-176* | 6 | 46,569 | Kan^R^, Tgc^S^ | Kan^S^, Tgc^R^ | Kan^R^, Tgc^R^ | 3.81 × 10^-4^ |
| SWHEFF_67 | *TEM-176*; *aph(3')-I*; *sul3*; *aadA*; *floR* | 5 | 47,888 | Kan^R^, Tgc^S^ | Kan^S^, Tgc^R^ | Kan^R^, Tgc^R^ | 3.54 × 10^-4^ |
| STEFF_10 | *aph(3')-I*; *catA* | 2 | 125,749 | Kan^R^, Tgc^S^ | Kan^S^, Tgc^R^ | Kan^R^, Tgc^R^ | 7.09 × 10^-6^ |
| STLEFF_38 | *aph(3')-I*; *qnrS*; *floR* | 3 | 39,362 | Kan^R^, Tgc^S^ | Kan^S^, Tgc^R^ | Kan^R^, Tgc^R^ | 1.28 × 10^-5^ |
| STEFF_16 | *TEM-176*; *floR*; *tetA*; *qnrS*; *dfrA14*; *aph(3')-I* | 6 | 46,569 | Tet^R^, Gen^S^ | Tet^S^, Gen^R^ | Tet^R^ + Gen^R^ | 9.60 × 10^-4^ |
| STEFF_21 | *tetA*; *dfrA12*; *aadA*; *sul1*; *mphA*; *aph(3')-I*; *sul2*; chloramphenicol exporter; *catA*; *sul2*; chloramphenicol exporter; *catA*; *qnrS* | 13 | 160,169 | Tet^R^, Gen^S^ | Tet^S^, Gen^R^ | Tet^R^ + Gen^R^ | 4.34 × 10^-4^ |
| STEFF_12 | *TEM-1*; *OXA-9*; *aadA*; *aac(6')-I*; *tetA*; *qnrS*; chloramphenicol exporter | 7 | 82,001 | Tet^R^, Gen^S^ | Tet^S^, Gen^R^ | Tet^R^ + Gen^R^ | 8.10 × 10^-7^ |
| STLIN_8 | *dfrA5*; *floR*; *tetA*; *TEM-176*; *aph(3')-I*; *qnrS* | 6 | 53,820 | Tet^R^, Gen^S^ | Tet^S^, Gen^R^ | Tet^R^ + Gen^R^ | 1.42 × 10^-5^ |
| SWHIN_100 | *tetA*; *TEM-1*; *sul2*; *sul1*; *aadA*; *dfrA12*; *mphA*; *aph(3')-I*; chloramphenicol exporter | 9 | 173,773 | Tet^R^, Gen^S^ | Tet^S^, Gen^R^ | Tet^R^ + Gen^R^ | 7.28 × 10^-5^ |

^a^ Antibiotic resistance genes are shown for the target conjugative plasmid in each donor bacteria.

^b^ Each donor selective resistance phenotype (i.e., Kan^R^: Kanamycin resistance or Tet^R^: Tetracycline resistance) is encoded in the target conjugative plasmid. Antibiotic concentration: Kanamycin (50 mg/L); Tigecycline (10 mg/L); Tetracycline (20 mg/L); Gentamicin (50 mg/L).

^c^ Conjugation frequencies are given as the number of transconjugants per recipient bacteria.

**Supplementary Figures**

**

** Supplementary Figure 1. The schematic view of the genetic constitution of all the identified conjugative plasmids using Plascad.


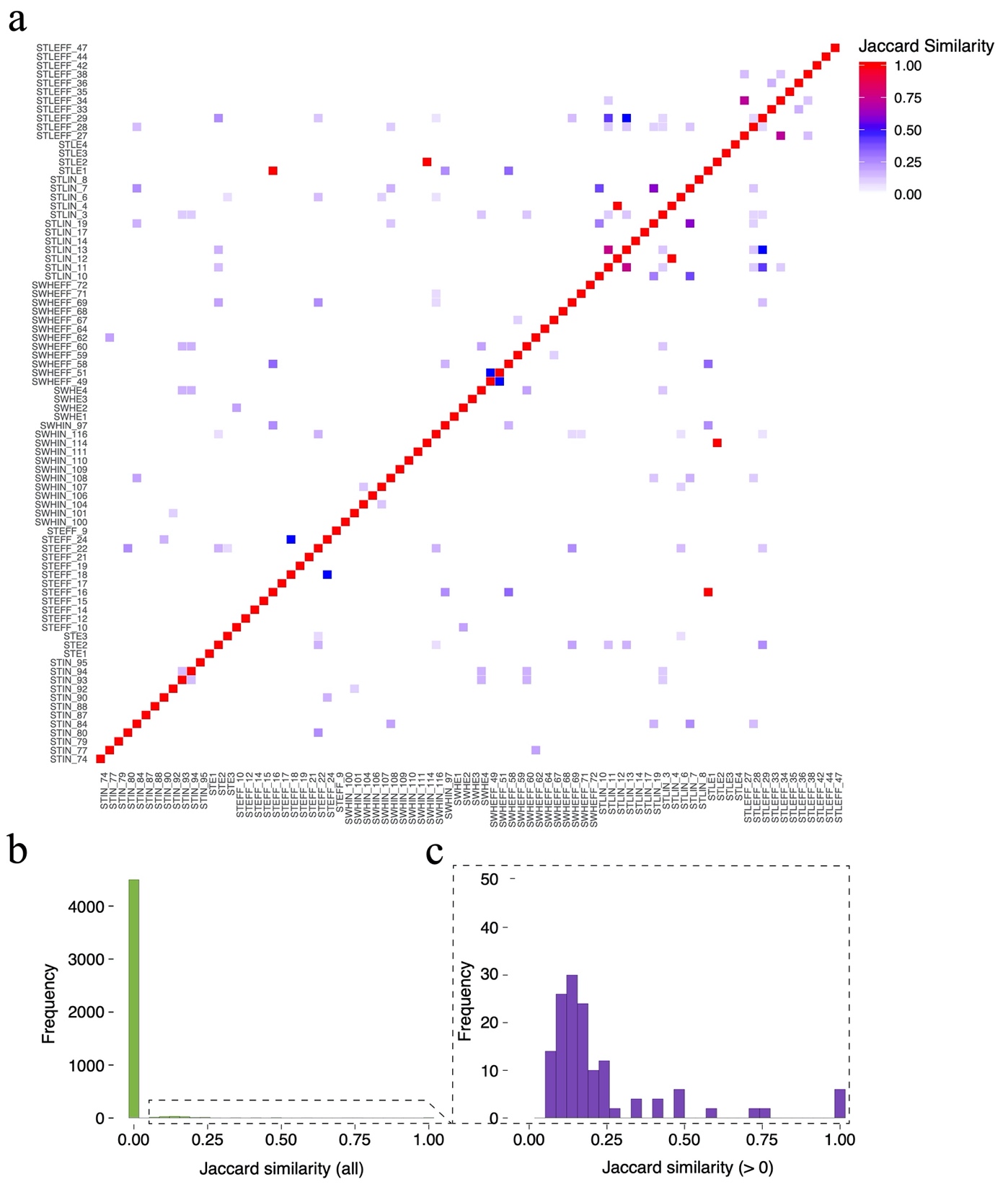


Supplementary Figure 2. Differences in plasmid composition among WWTPs-associated MDR isolates. a, Pairwise similarity of plasmid composition (measured by Jaccard index). b, Jaccard index frequency distribution (including all Jaccard similarity: 0-1). c, Zoom view of Jaccard index (Jaccard similarity above 0) frequency distribution.


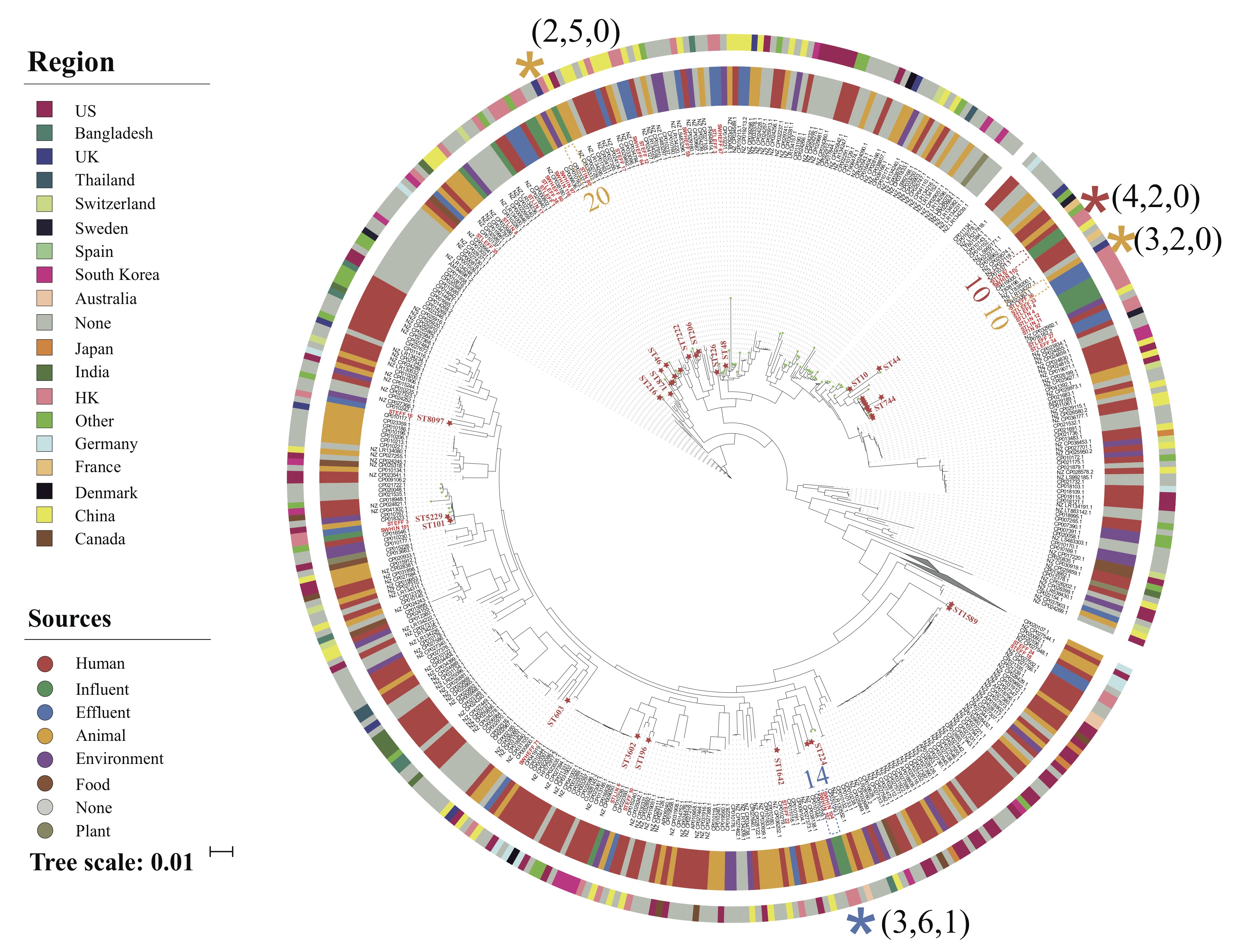


Supplementary Figure 3. Phylogeny of a total of 623 *Escherichia flexneri* (WWTPs: 37; Public: 586) based on core genome SNPs. The inner colored ring indicates the isolation sources, and the outer colored ring indicates the geographical location of isolates. Our WWTP isolates are shown in red, with MLST information included. Green stars adjacent to the STs names indicate global isolates with the same STs as the WWTP isolates. The closely related isolates (defined as differing by < 30 core genome SNPs) between WWTPs and humans (red asterisks)/animals (yellow asterisks), and within WWTPs (blue asterisks) are highlighted, with the adjacent number showing core genome SNPs. The tree of *Escherichia flexneri* is graphically simplified by collapsing several clades containing genomes into a single leaf.


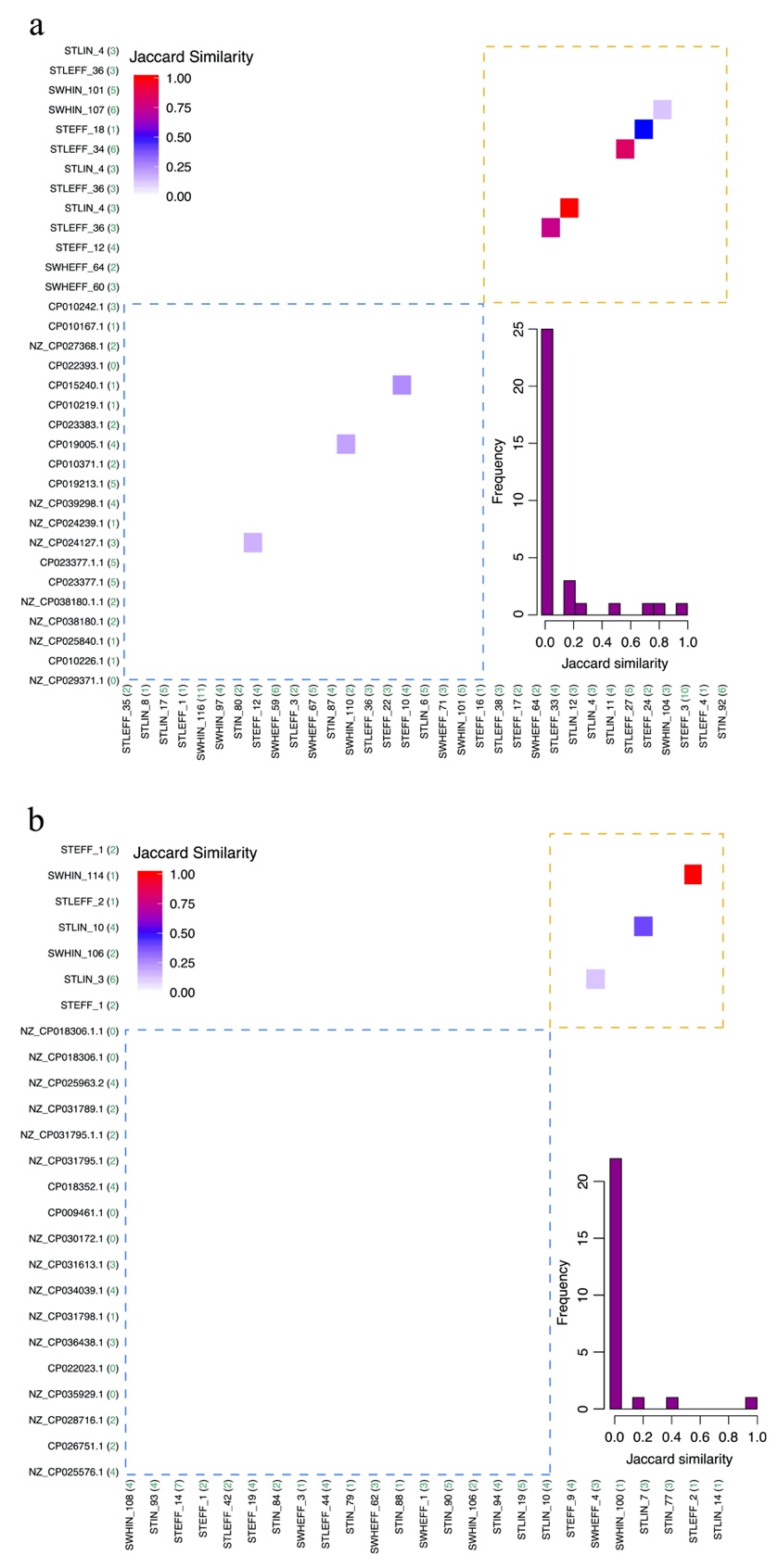


Supplementary Figure 4. Distinct plasmid profiles between WWTPs-associated isolates (a: *E. flexneri*; b: *K. pneumoniae*) and their closet human/anima-associated relatives in the public database. Comparisons of plasmid profiles between WWTPs-associated isolates and their closest human/animal-associated relatives are highlighted in blue dash rectangles, while those comparisons between WWTPs-associated isolates are highlight in yellow dash rectangles. The green number in brackets shows the number of plasmids in the corresponding isolate and the barcharts in the lower right corner indicate Jaccard index frequency distribution.

**
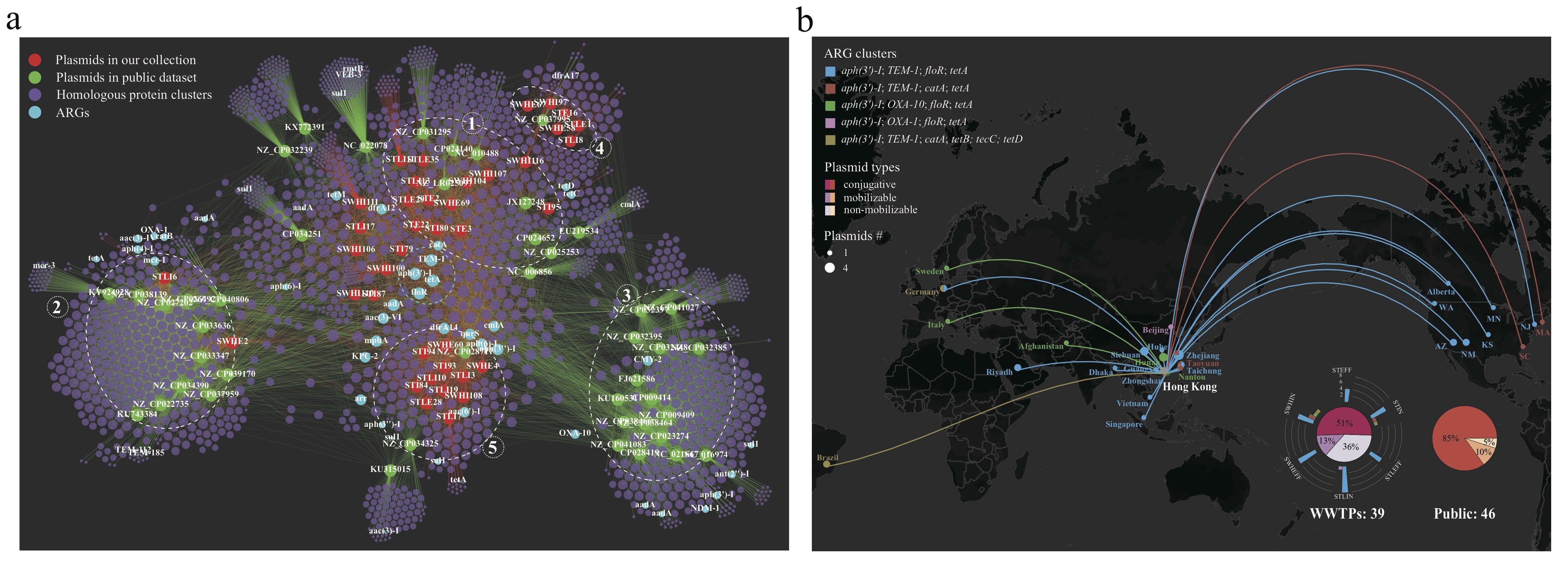
**Supplementary Figure 5, a, Bipartite network of potential horizontal transfer of ARGs among plasmids. Five major plasmid clusters are indicated by white dotted circles with cluster numbers (1-5), which match the five lineages identified in Figure. 5. The most widely transferred ARG cluster (i.e., *aph(3’)-I*, *bla*_TEM-1_, *floR*, and *tetA*) is highlighted by vivid cyan dotted circle in the center of the network. b, Global distribution of the ARG clusters. The size of each node indicates the number of plasmids carrying the corresponding ARG clusters, and the colored lines denote the corresponding clusters. Pie charts are plotted showing the summary of all the plasmids involved.


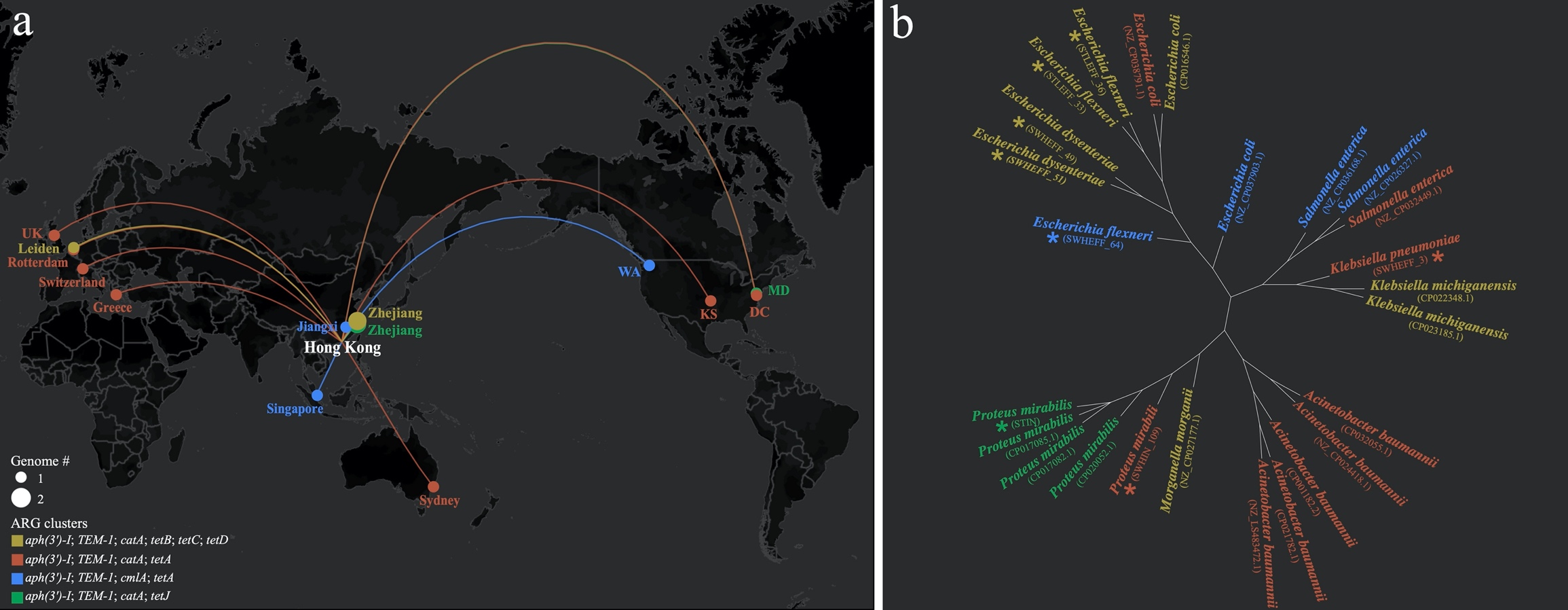


Supplementary Figure 6. Potential horizontal transfer of ARG clusters conferring resistance to four antibiotics across diverse chromosome backgrounds (25 in total) and ecological barriers**.** a, Global spread of the ARG clusters. The size of each node indicates the number of bacterial chromosomes carrying the corresponding ARG clusters, and the colored lines denote the corresponding clusters. b, Maximum likelihood phylogenetic analysis of a total of 25 genomes (WWTPs: 8; Public: 27) carrying the shared ARGs clusters. The colored names denote the corresponding ARG clusters.


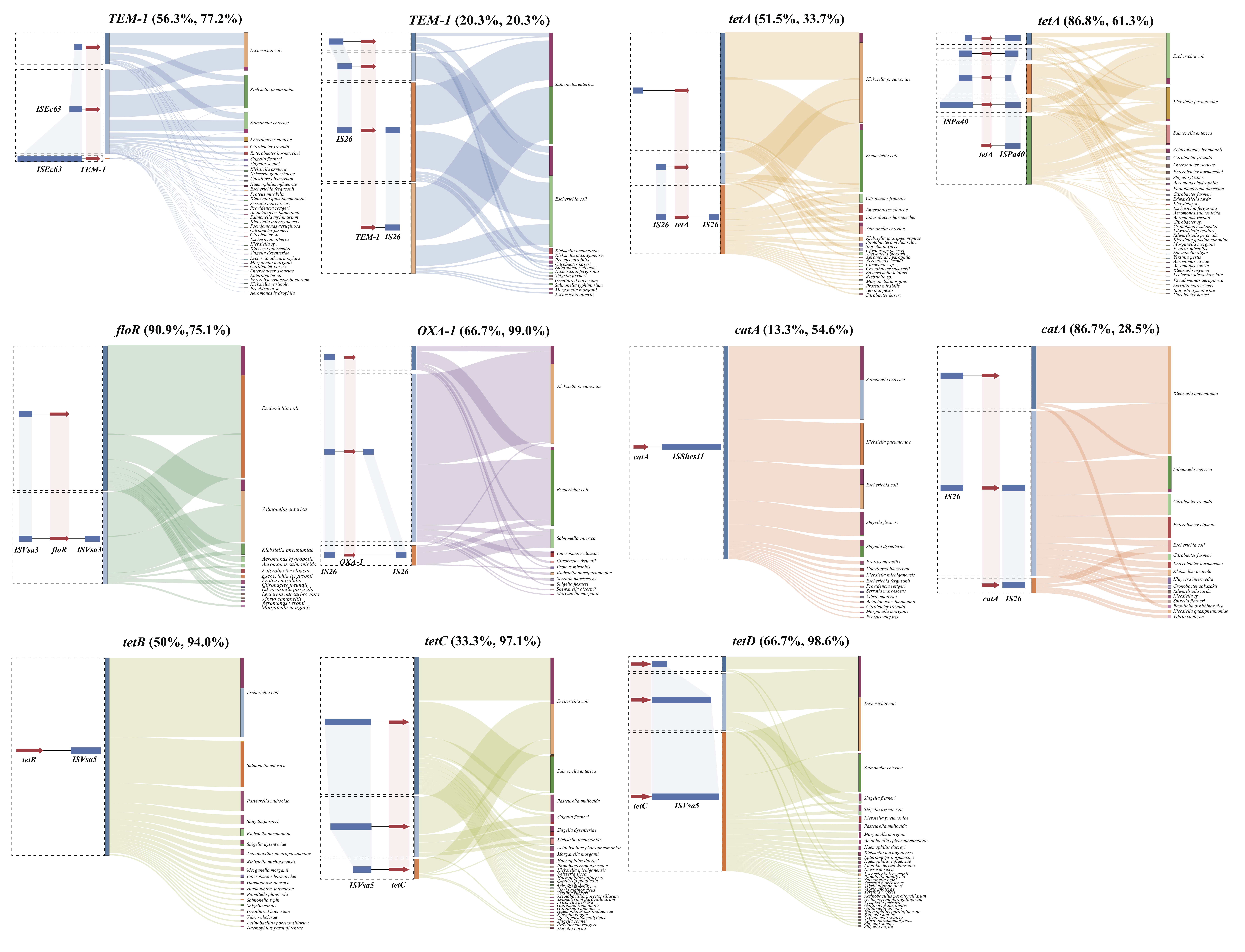


Supplementary Figure 7. ISs play a critical role in expanding the transfer range of ARGs. Evidence for ISs-associated ARG transfer across different genetic contexts between WWTPs and public datasets. The plasmid-borne ARGs (i.e., *bla*_TEM-1_, *tetA*, *floR*) and those (i.e., *bla*_OXA-1_, *catA*, *tetB*, *tetC*, and *tetD*) that are functionally related to the four selective antibiotics in the WWTPs are shown in this figure.

**
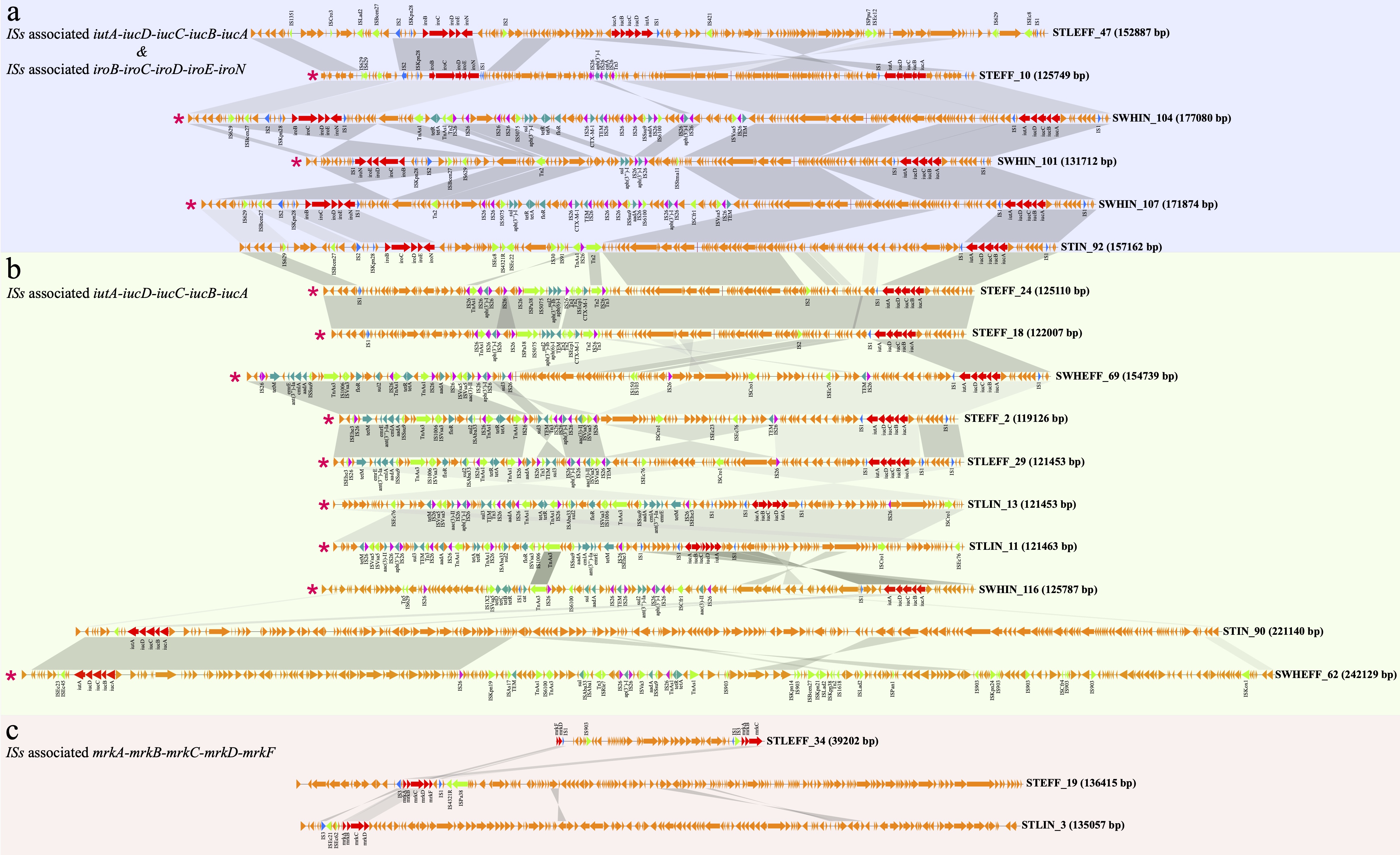
**

Supplementary Figure 8. VFs are closely associated with ISs in plasmids. a, Genetic maps for the co-localized hypervirulence determinants (*iuc* and *iro* loci). b, Plasmids with only *iuc* loci. c, Plasmids with *mrk* loci. Genes that make up all the VFs are highlighted with red arrows. ISs and ARGs are indicated (IS1, IS2, ISKpn28, IS3, and IS26 are specified and other ISs are colored green). MDR-hypervirulent plasmids are highlighted with dark pink asterisk on the left. Grey shading indicates homology blocks sharing between different plasmids.


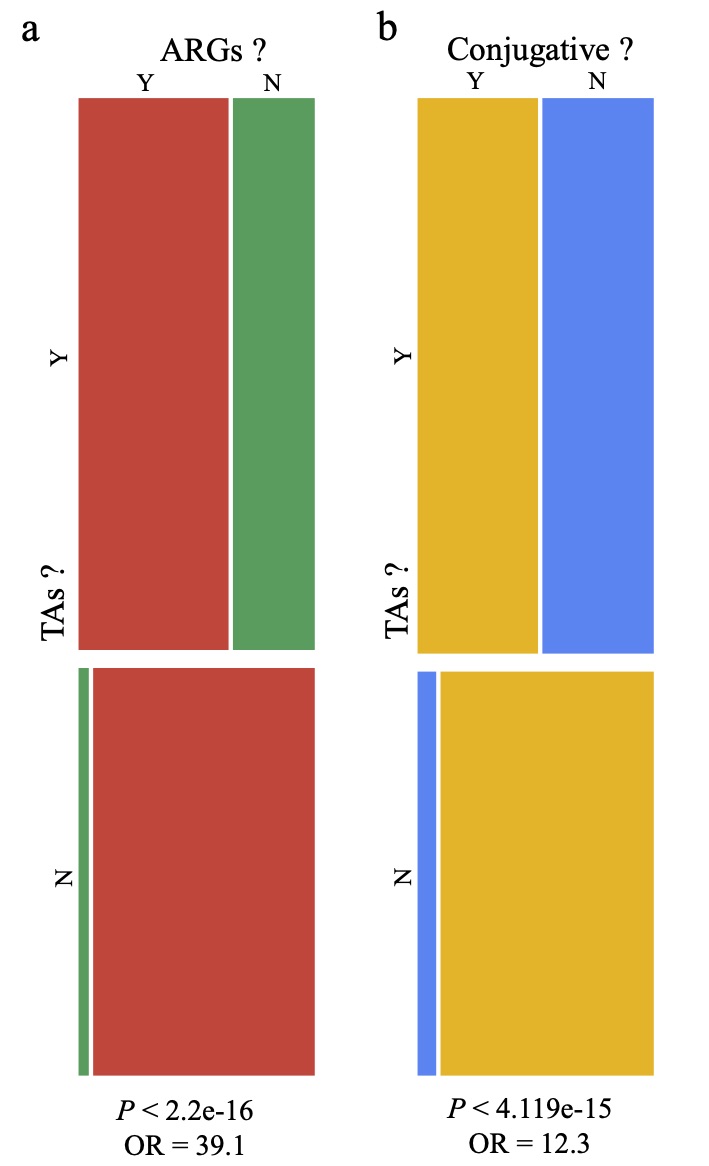


Supplementary Figure 9. Mosaic plots showing the relationship between toxin-antitoxin systems, ARGs and categories of plasmids by Fisher’s exact test. The area of each box is proportional to the number of elements in that group.


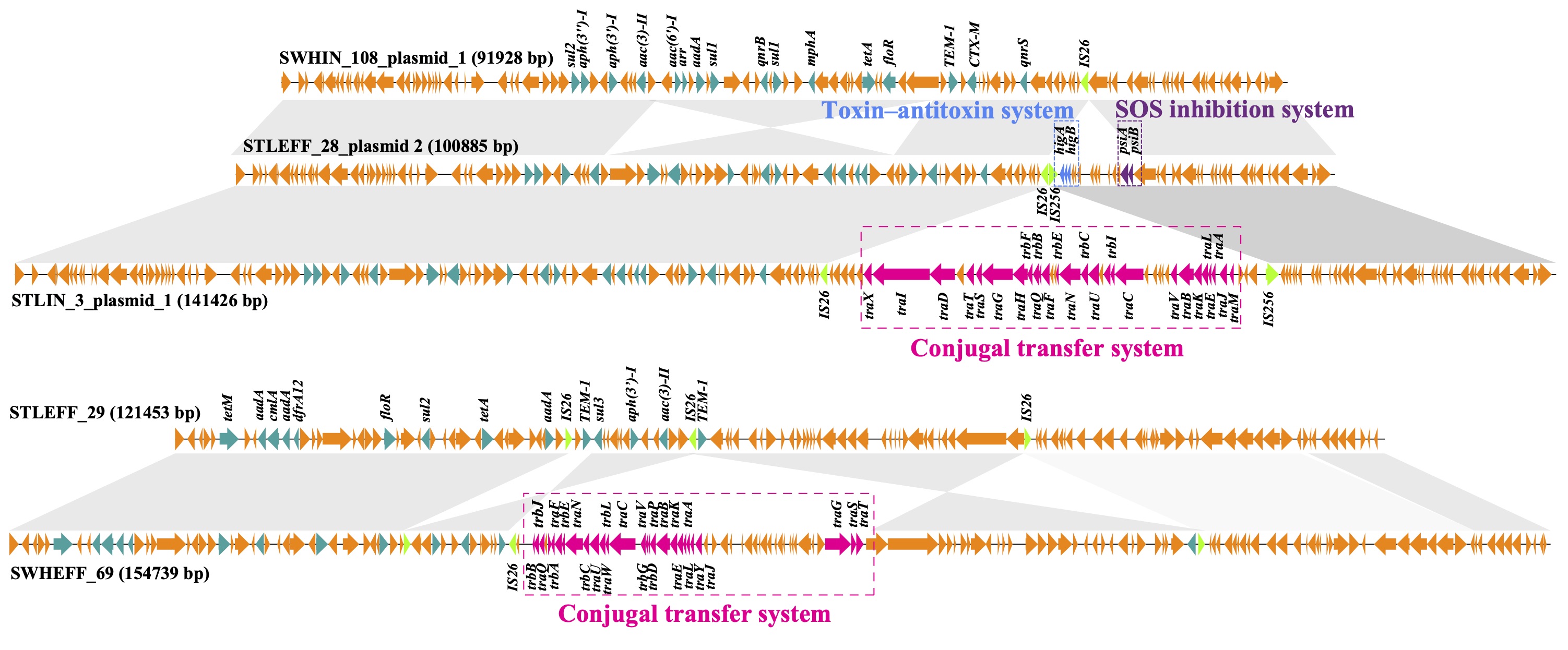


Supplementary Figure 10. ISs play an important role for plasmid evolution by maintaining the plasticity necessary to balance the cost of plasmid maintenance. Comparative analysis of plasmid contents reveals dynamic evolution of genes encoding toxin-antitoxin system, plasmid SOS inhibition system and conjugal transfer system due to the flanking ISs. ARGs and ISs are indicated (IS26 and IS256 are specified in green). Grey shading indicates homology blocks sharing between different plasmids.
